# Multitasking of Hsp70 chaperone in the biogenesis of bacterial functional amyloids

**DOI:** 10.1038/s42003-018-0056-0

**Published:** 2018-05-31

**Authors:** Shinya Sugimoto, Ken-ichi Arita-Morioka, Akari Terao, Kunitoshi Yamanaka, Teru Ogura, Yoshimitsu Mizunoe

**Affiliations:** 1 Department of Bacteriology, The Jikei University School of Medicine, 3-25-8 Nishi-Shimbashi, Minato-Ku, Tokyo, 105-8461 Japan; 2 Jikei Center for Biofilm Science and Technology, The Jikei University School of Medicine, 3-25-8 Nishi-Shimbashi, Minato-Ku, Tokyo, 105-8461 Japan; 3 Department of Molecular Cell Biology, Institute of Molecular Embryology and Genetics, Kumamoto University, 2-2-1 Honjo, Chuo-Ku, Kumamoto, 860-0811 Japan; 4 Advanced Science Research Center, Fukuoka Dental College, 2-15-1 Tamura, Sawara-Ku, Fukuoka, 814-0193 Japan

## Abstract

Biofilms are intricate communities of microorganisms embedded in a self-produced matrix of extracellular polymer, which provides microbes survival advantages in stressful environments and can cause chronic infections in humans. Curli are functional amyloids that assemble on the extracellular surface of enteric bacteria such as *Escherichia coli* during biofilm development and colonization. The molecular chaperone DnaK, a bacterial Hsp70 homologue, promotes curli biogenesis via unknown mechanism(s). Here we show that DnaK increases the expression of CsgA and CsgB—the major and minor structural components of curli, respectively—via a quantity and quality control of RpoS, a stationary phase-specific alternative sigma factor regulating bacterial transcription, and CsgD, the master transcriptional regulator of curli formation. DnaK also keeps CsgA and CsgB in a translocation-competent state by binding to their signal peptides prone to aggregation. Our findings suggest that DnaK controls the homoeostasis of curli biogenesis at multiple stages to organize the biofilm matrix.

## Abstract (web summary)

Shinya Sugimoto et al. demonstrate how molecular chaperone DnaK regulates biofilm formation through the production of curli, which anchor enteric bacteria to the biofilm. This finding provides mechanistic insights into the development of anti-biofilm agents as antibiotics.

## Introduction

Biofilms are highly organized communities of microbes that form on biotic and abiotic surfaces and can cause chronic or fatal infectious diseases in humans^1^. In enteric bacteria, extracellular amyloids known as curli are the major extracellular polymeric substances that modulate biofilm organization and colonization by adhering to surfaces and anchoring cells to the biofilm^2,3^. Similarly, other microbial extracellular amyloids are also important for adhesion of microbes to the host surface, which can lead to persistent infections by opportunistic pathogens such as *Pseudomonas aeruginosa* and *Staphylococcus aureus*^4,5^. Curli are composed of unbranched, highly aggregative, β-sheet-rich filaments with a diameter of 4–6 nm that are resistant to protease digestion and chemical denaturation, and are biochemically and structurally similar to pathogenic amyloid fibrils associated with neurodegeneration in Alzheimer’s, Parkinson’s, Huntington’s, and prion diseases^6^.

Unlike pathogenic amyloids that result from protein misfolding, curli are generated via a secretory nucleation-precipitation mechanism or the type VIII secretion system^7^; in *Escherichia coli*, seven proteins encoded by two operons—curli-specific genes *BAC* (*csgBAC*) and *DEFG* (*csgDEFG*)—regulate curli expression, export, and assembly^8^. The major curli subunit CsgA has three domains: an N-terminal signal peptide (residues 1–20), the CsgG-recognition sequence (residues 21–42), and five imperfect amyloidogenic repeats (R1–5, residues 43–151) containing an S-X_5_-Q-X-G-X_2_-N-X_5_-Q motif^9,10^. Following translocation across the cytoplasmic membrane through the Sec translocon, the signal peptide is proteolytically cleaved^3^, yielding a ~13-kDa mature CsgA subunit that is exported across the outer membrane in a CsgG-dependent manner^11,12^. Exported soluble CsgA is nucleated by CsgB, the minor curli subunit^13^, to induce amyloid assembly. Like CsgA, CsgB contains three domains: an N-terminal signal peptide (residues 1–21), CsgG-recognition sequence (residues 22–44), and five imperfect amyloidogenic repeats (R1–5, residues 45–151)^10^ and can form amyloid fibrils in vitro^13^. Recently, the periplasmic chaperone-like protein CsgC was shown to bind directly to CsgA in vitro and prevent premature aggregation^14^. The *csgDEFG* operon encodes CsgD, a master transcriptional regulator of curli biogenesis that acts as a positive regulator of the *csgBAC* operon^15^, the periplasmic accessary protein CsgE^16^, the extracellular accessory protein CsgF^17^, and the outer membrane curli-specific translocation channel CsgG^18^. CsgE targets CsgA to CsgG for secretion and can inhibit CsgA amyloid assembly in vitro^16^. CsgF is exported and is required for the specific localization and/or nucleation activity of CsgB. However, the quality control of this amyloidogenic protein prior to its translocation to the periplasm is poorly understood.

Maintaining protein homoeostasis (proteostasis) is essential for diverse cellular activities in all life forms. Molecular chaperones prevent protein misfolding and aggregation, typically by shielding exposed hydrophobic surfaces in denatured and non-native proteins^19^. DnaK, the major bacterial heat-shock protein (Hsp)70, is a constitutively expressed stress-inducible chaperone in *E. coli* that functions in the folding of newly synthesized proteins, refolding of denatured and aggregated proteins, and protein transport and quality control in cooperation with DnaJ and GrpE^20^. DnaK has a 45-kDa N-terminal nucleotide-binding domain and a 25-kDa C-terminal substrate-binding domain that are connected by a short peptide linker^21^. In its ATP-bound state, DnaK shows low affinity for substrates; however, the ADP-bound state has high substrate affinity and hence exhibits slow rates of substrate binding and release. The DnaK ATP hydrolysis cycle is controlled by the ATPase-stimulator DnaJ and the nucleotide exchange factor GrpE^22^. The cytoplasmic chaperones DnaK and Hsp33 were previously shown to inhibit CsgA amyloid assembly in vitro^23^. In addition, we recently reported that DnaK plays an important role in curli-dependent biofilm formation and is a potential target for anti-biofilm compounds^24^. However, the regulation of curli biosynthesis by DnaK and the contribution of other protein quality control systems to this process are unclear.

To address these issues, in this study we systematically screen molecular chaperones and proteases to investigate their roles in curli biogenesis and biofilm formation. DnaK is the central component in this process that regulates the quantity and quality of the transcriptional regulators RpoS and CsgD to modulate *csgBAC* and *csgDEFG* expression. CsgA and CsgB translocation across the cytoplasmic membrane is dependent on DnaK chaperone activity. Finally, we present evidence that the interaction between DnaK and the N-terminal signal peptides of these amyloidogenic proteins facilitates their translocation in vivo. Our findings indicate that DnaK has a multifunctional role in bacterial amyloid biogenesis.

## Results

### DnaK is involved in curli-dependent biofilm formation

**Fig. 1:**
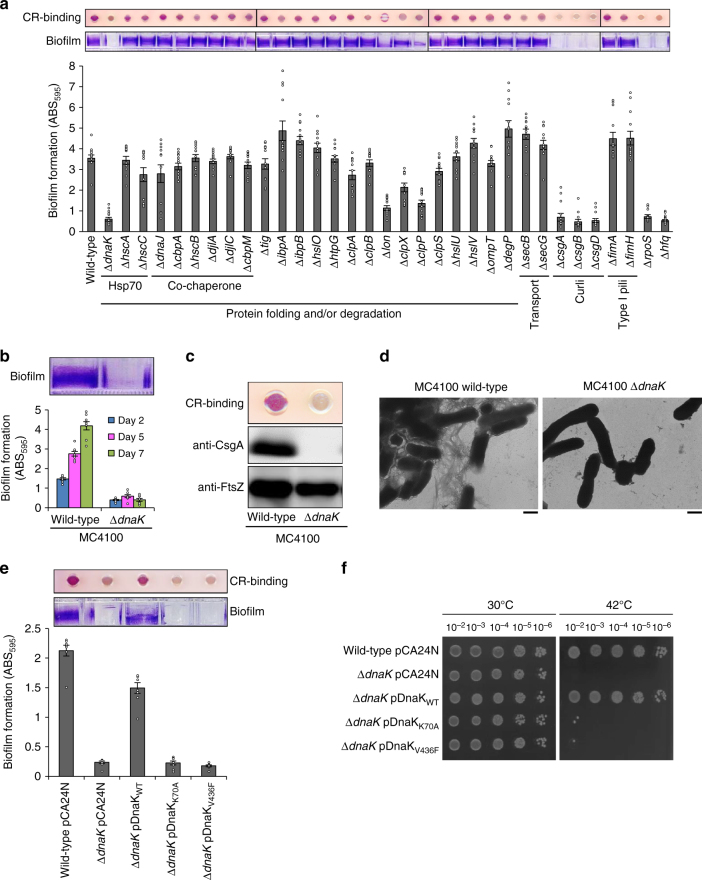
DnaK is important for curli-dependent biofilm formation. **a** Curli production in indicated *E. coli* strains (Keio collection) was analysed with the CR-binding assay (upper panel). Biofilms formed in a 96-well polystyrene plate were stained with crystal violet (middle panel). The bottom graph shows the quantification of biofilm biomass. **b** Biofilm formation of other strains. The upper panel shows 7 days biofilms; biomasses were quantified after 2, 5, and 7 days of incubation. **c** Curli production by indicated *E. coli* strains was analysed with the CR-binding assay and immunoblotting using anti-CsgA antibody. Curli fibrils were depolymerized to CsgA monomers by applying hexafluoroisopropanol. FtsZ was detected as a loading control. **d** Extracellular structures of indicated *E. coli* strains were analysed by transmission electron microscopy. Scales, 500 nm. **e** Curli production and biofilm formation of the indicated strains were analysed with CR-binding assay and by crystal violet staining. **f** Complementation assay for evaluating the recovery of the growth defect at high temperature in Δ*dnaK*. Experiments were repeated at least three times. Means with standard errors and data plots are shown. Full-size scans of immunoblots are shown in Supplementary Fig. 2

We investigated the protein quality control systems that are required for curli-dependent biofilm organization using a subset of the Keio collection, an *E. coli* single-gene-knockout mutant library^25^. All strains were grown in YESCA medium at 30 °C. Knockouts of *csg* genes (*csgA*, *csgB*, and *csgD*) but not of *fim* genes (*fimA* and *fimH*) related to type I pili resulted in a reduction of biofilm organization (Fig. 1a). Deficiency in genes responsible for flagella construction did also not affect biofilm formation (Supplementary Fig. 1). Under the conditions used in this study, curli—but neither type I pili nor flagella—was required for biofilm formation as previously reported^24,26^. In this study, we focused on the conditions that promote curli-dependent biofilm formation.

Among the genes associated with proteostasis, deletion of *dnaK* drastically reduced *E. coli* BW25113 biofilm biomass (Fig. 1a) and curli production, as determined with the Congo Red (CR) binding assay (Fig. 1a) and by immunoblotting and transmission electron microscopy^24^. Similar results were observed in another genetic background (Fig. 1b–d and Supplementary Fig. 2). Deletion of the *lon*, *clpX*, or *clpP* gene moderately reduced biofilm biomass, whereas loss of genes encoding cytoplasmic chaperones and proteases, periplasmic chaperones, and membrane proteases had no effect on biofilm formation or curli production (Fig. 1a and Supplementary Fig. 1). We therefore focused on the molecular mechanism(s) underlying curli biogenesis regulated by DnaK.

Expression of a plasmid-borne wild type DnaK (DnaK_WT_) complemented the biofilm formation and curli production deficiencies of the ∆*dnaK* strain (Fig. 1e). We then generated two DnaK mutants: one with a Lys-70-Ala substitution in the nucleotide-binding domain (DnaK_K70A_), which produced a defective ATPase activity^27^; and one with a Val-436-Phe substitution in the substrate-binding domain (DnaK_V436F_), which decreased substrate affinity^28^. Neither DnaK_K70A_ nor DnaK_V436F_ was able to rescue the thermosensitivity of the ∆*dnaK* strain (Fig. 1f) and to restore the deficiencies in biofilm formation and curli production (Fig. 1e), indicating that DnaK is required for regulation of curli biosynthesis and biofilm formation.

### DnaK modulates the expression of *csg* genes

**Fig. 2:**
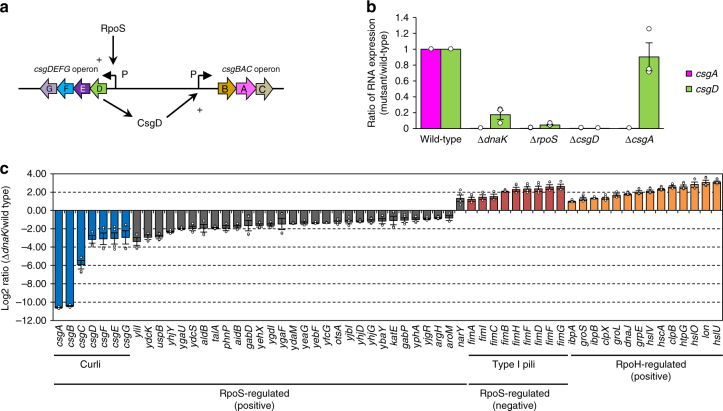
Deletion of the *dnaK* gene alters *csg* gene expression. **a** Simplified schematic diagram of *csg* regulons. RpoS positively regulates the transcription of the *csgDEFG* operon. The master regulator CsgD induces the *csgBAC* operon. **b** *csgA* and *csgD* mRNA levels in indicated strains were analysed by real-time-PCR. **c** Microarray data showing log_2_ fold changes in the expression levels of genes positively regulated by RpoS^66^. The expression of *fim* genes associated with the production of type I pili is negatively regulated by RpoS^31^; the expression of genes positively regulated by RpoH is also shown^32^. All analyses were repeated at least three times and average values with standard errors and plots of quantification data are shown

To investigate whether the expression of *csg* genes is affected by loss of DnaK, we evaluated the transcript levels of *csgA* and *csgD* encoded by the *csgBAC* and *csgDEFG* operons, respectively (Fig. 2a), by real-time PCR analysis. The expression of the *csgDEFG* and *csgBAC* operons is controlled by diverse factors^29^; for instance, the alternative sigma factor RpoS positively regulates *csgDEFG* transcription (Fig. 2a)^8,30^, whereas CsgD—a master regulator of curli synthesis—directly activates *csgBAC* transcription (Fig. 2a)^8,15^. We therefore used ∆*rpoS* and ∆*csgD* mutants as controls in this analysis. The *csgA* and *csgD* transcripts were downregulated in ∆*dnaK* relative to the wild type (Fig. 2b). A microarray analysis confirmed that *csg* gene expression was decreased. Importantly, the expression of genes regulated by RpoS was also reduced in the ∆*dnaK* strain as compared to the wild type (Fig. 2c and Supplementary Data 1). In contrast, *fim* genes—which are associated with type I pili and are negatively regulated by RpoS^31^—showed the opposite trend (Fig. 2c). The transcript levels of genes that are positively regulated by RpoH^32^ were also increased in ∆*dnaK* as compared to the wild type (Fig. 2c), which is consistent with the previously reported increase in RpoH level in the ∆*dnaK* strain^24^. In addition, the activity of catalase—whose expression is modulated by RpoS—was reduced in ∆*dnaK* (Supplementary Fig. 3a), and this could not be restored by overexpressing DnaK_K70A_ or DnaK_V436F_ (Supplementary Fig. 3b). These results indicate that RpoS quality or quantity is severely compromised by loss of DnaK function.

### DnaK influences RpoS quantity and quality

**Fig. 3:**
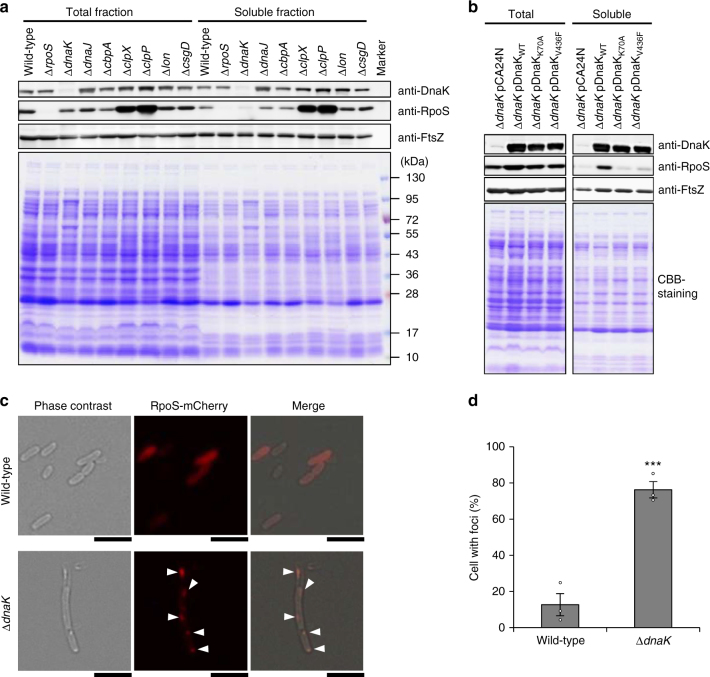
DnaK is required for the correct folding of RpoS. **a** Total and soluble fractions of indicated strains were separated by SDS-PAGE and stained with CBB. Proteins were detected by immunoblotting using indicated antibodies. FtsZ served as the loading control. Molecular masses are indicated to the right of the panel. **b** Complementation of *dnaK* deletion with indicated plasmids. Immunoblotting was performed as shown in panel **a**. **c** RpoS-mCherry was expressed from the plasmid pRpoS-mCherry in BW25113 wild-type and its isogenic Δ*dnaK* mutant. Arrowheads indicate cells with RpoS-mCherry foci. Scale bars, 5 μm. **d** Percentages of cells with foci in panel **c** were calculated and are shown as mean ± standard errors of three experiments (*n* = 821). Average values of three experiments are shown as plots. ***P < 0.001. Full-size scans of immunoblots are shown in Supplementary Figs. 4 and 5

We examined RpoS quantity and quality in total and soluble fractions of *E. coli* cell lysates by immunoblotting. RpoS is degraded by the ATP-dependent protease ClpXP; accordingly, the level of RpoS was higher in ∆*clpX* and ∆*clpP* mutants than in the wild-type strain (Fig. 3a and Supplementary Fig. 4). In contrast, RpoS level was lower in ∆*dnaK* than in wild-type cell lysates (Fig. 3a and Supplementary Fig. 4). This is in agreement with a previous observation that DnaK protects RpoS from proteolysis^33^. Soluble RpoS was drastically reduced in ∆*dnaK* as compared to the wild type (Fig. 3a and Supplementary Fig. 4); this was rescued by DnaK_WT_ but not by DnaK_K70A_ or DnaK_V436F_ (Fig. 3b and Supplementary Fig. 5). A cytological analysis revealed that RpoS-mCherry fusion proteins aggregated in ∆*dnaK* but not in wild-type cells (Fig. 3c, d and Supplementary Fig. 6), implying that DnaK controls RpoS quantity and quality. In addition, the mutant strains lacking DnaJ and CbpA—which are co-chaperones that stimulate the ATPase activity of DnaK—showed the similar RpoS solubility compared with wild type (Fig. 3a and Supplementary Fig. 4). Biofilm formation and curli production were also unaffected in ∆*dnaJ* and ∆*cbpA* mutants (Fig. 1a), suggesting two possibilities: (i) in addition to DnaJ and CbpA, the third DnaJ-family protein DjlA is required for curli biogenesis or (ii) these three DnaJ-family proteins that cooperate with DnaK as co-chaperones is not required in this process. These possibilities will be addressed in future.

### DnaK promotes the correct folding of CsgD

**Fig. 4:**
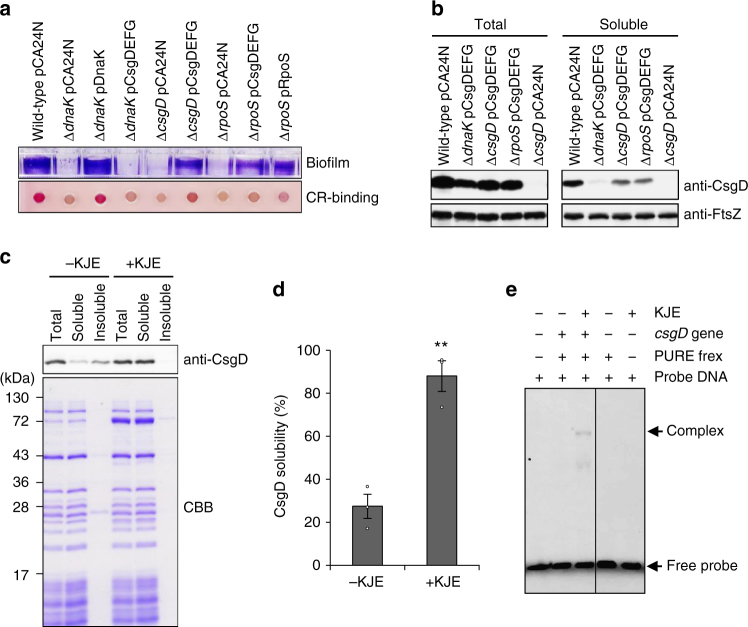
DnaK contributes to CsgD folding. **a** Biofilm formation and curli production by indicated strains were analysed as shown in Fig. 1. **b** Protein folding states of CsgD were analysed by immunoblotting. FtsZ served as a control. **c** CsgD was synthesized in a cell-free translation system in the absence and presence of DnaK-DnaJ-GrpE (KJE). Proteins were separated into soluble and insoluble fractions by centrifugation and CsgD was detected by immunoblotting. Molecular masses are indicated to the left of the panel. **d** Solubility of CsgD was quantified based on the intensity of protein bands shown in panel **c**. Experiments were repeated at least three times and average values with standard errors and data plots are shown. **P < 0.01. **e** DNA-binding activity of CsgD generated in the cell-free translation system was examined by gel-shift assay. The double-stranded DNA fragment harbouring the *csgB* promoter region was probed with Alexa 488 and incubated with the indicated reaction mixtures. +, presence; −, absence. Full-size scans of immunoblots are shown in Supplementary Figs. 8 and 9

Our data from real-time PCR and microarray analyses indicated that expression of the *csgBAC* operon was decreased to a greater degree than that of the *csgDEFG* operon upon deletion of *dnaK* (Fig. 2b, c), suggesting that the CsgD levels fell below their active concentration and/or that CsgD quality was compromised in the Δ*dnaK* strain. We attempted to investigate biofilm formation and curli production by introducing a CsgD-expression plasmid into the ∆*csgD* strain; however, this did not complement *csgD* deficiency (Supplementary Fig. 7), likely due to lower levels of the *csgEFG* genes located downstream of *csgD* on the chromosome. We therefore constructed a *csgDEFG* co-expression plasmid (pCsgDEFG) that was able to restore biofilm formation and curli production in ∆*csgD*; this was not the case for pCsgD*EFG, which co-expressed CsgEFG and an inactive form of CsgD lacking the DNA-binding domain (Fig. 4a and Supplementary Fig. 7). The pCsgD*EFG plasmid complemented ∆*csgE*, ∆*csgF*, and ∆*csgG* (Supplementary Fig. 7), indicating that pCsgDEFG and pCsgD*EFG were functional in the respective mutant strains and that active CsgD, CsgE, CsgF, and CsgG were required in the ∆*csgD* strain for curli production. pCsgDEFG partially rescued biofilm formation and curli production in the ∆*rpoS* strain (Fig. 4a), suggesting that RpoS is not essential but supportive for expression of the *csgBAC* operon at least under the conditions of this study, as previously reported^30,34^.

Importantly, the ∆*dnaK* strain harbouring pCsgDEFG did not produce curli or form biofilm. In these cells, CsgD was synthesized but its soluble form was hardly detected by immunoblotting (Fig. 4b and Supplementary Fig. 8). This is consistent with results from a previous screen of the DnaK-binding proteome that reported a requirement for DnaK in CsgD folding in vivo^35^.

We next addressed whether DnaK directly modulates CsgD folding and activity using a cell-free translation system (i.e., the Protein Synthesis Using Recombinant Elements [PURE] System)^36^. We found that a DnaK chaperone system consisting of DnaK, DnaJ, and GrpE (KJE) was required for CsgD folding, as evidenced by its solubility (Fig. 4c, d and Supplementary Fig. 9). This is in agreement with a previous analysis of protein folding in *E. coli* using the PURE System^37^. In vitro-synthesized and KJE-assisted soluble CsgD was capable of binding to the *csgB* promoter (Fig. 4e), indicating that KJE contributes to the productive folding of CsgD.

### DnaK is required for the transport of CsgA

**Fig. 5:**
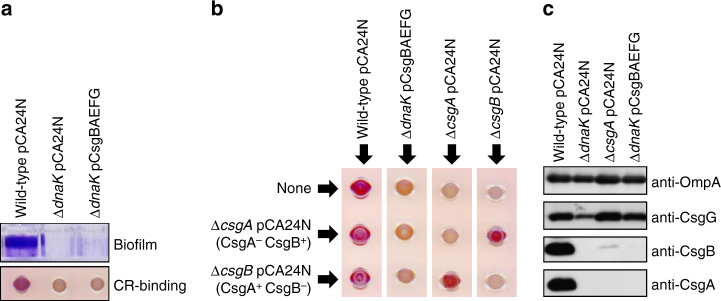
DnaK is involved in the extracellular transport of CsgA and CsgB. **a** Biofilm formation and curli production in indicated strains were examined as in Fig. 1. **b** Interbacterial complementation assay performed using indicated strains. Curli production was analysed with the CR-binding assay. **c** Membrane localization of CsgA, CsgB, and CsgG was analysed by cell fractionation and immunoblotting. Hexafluoroisopropanol was used to depolymerize CsgA fibrils. Outer membrane-localized OmpA was detected to verify the reliability of the fractionation. Full-size scans of immunoblots are shown in Supplementary Fig. 10

In *E. coli*, secreted proteins are maintained in an export-competent state prior to their translocation across the cytoplasmic membrane. DnaK also participates in the export of several proteins, most likely by acting as a molecular chaperone^38,39^. We therefore investigated whether DnaK is involved in the transport of CsgA by expressing the pCsgBAEFG plasmid in ∆*dnaK* cells. The functionality of the plasmid was confirmed by the observation that it restored biofilm formation and curli production in ∆*csgA*, ∆*csgB*, ∆*csgE*, ∆*csgF*, and ∆*csgG* (Supplementary Fig. 7). However, pCsgBAEFG expression did not rescue curli production in the ∆*dnaK* strain (Fig. 5a).

We examined whether CsgA and CsgB are expressed extracellularly and at the cell surface, respectively, with the interbacterial complementation assay^3^. Extracellular soluble CsgA proteins produced by ∆*csgB* cells interacted with CsgB proteins on the surface of ∆*csgA* cells and assembled into curli amyloid fibrils, which was observable on CR-agar plates (Fig. 5b). However, the ∆*dnaK* CsgBAEFG^+^ strain did not produce curli even when mixed with both indicator strains, suggesting that neither CsgA nor CsgB was expressed at the correct subcellular locations (Fig. 5b). Cell fractionation and immunoblot analyses supported these results (Fig. 5c and Supplementary Figs. 10 and 11). Given that CsgA and CsgB were not detected by immunoblotting even after treatment with hexafluoroisopropanol, which can disassemble curli amyloid fibrils into CsgA and CsgB monomers, CsgA and CsgB production may be highly toxic in ∆*dnaK* cells, leading to their elimination during cultivation. In contrast, CsgG was detected in the membrane fraction of ∆*dnaK* cells and the protein level was restored to that of wild-type cells upon introduction of pCsgBAEFG (Fig. 5c and Supplementary Fig. 10), indicating that the plasmid was maintained in this strain and produced CsgG.

### CsgA aggregation depends on an N-terminal signal peptide

**Fig. 6:**
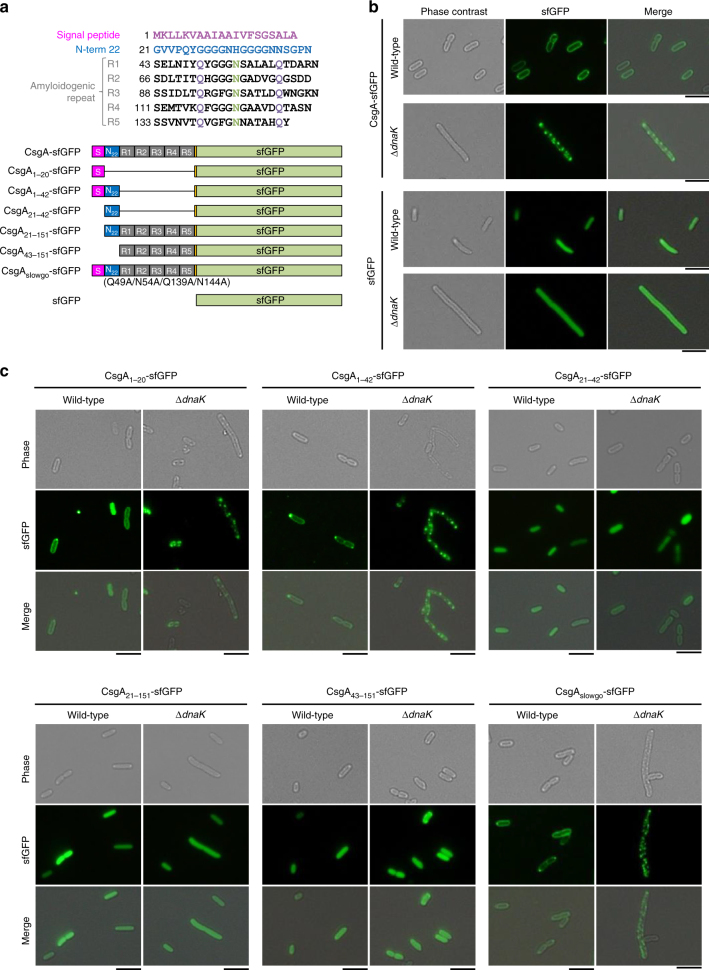
Visualization of CsgA transport to periplasm and intracellular aggregation in vivo. **a** Amino acid sequence of CsgA and domain structure of sfGFP-fused CsgA variants used in this study. Magenta letters and boxes indicate the signal peptide; blue letters and boxes represent the CsgG-recognition sequence. Amyloidogenic five repeat sequences (R1–5) are also shown. **b** CsgA-sfGFP and sfGFP were expressed from plasmids in the BW25113 wild-type strain and its isogenic Δ*dnaK* mutant. **c** CsgA-sfGFP derivatives were expressed as illustrated in panel **b**

Fluorescent protein fusions are useful for visualizing the subcellular localization and folding status of proteins in a cell. In this study, we used superfolder green fluorescent protein (sfGFP)^40^ for this purpose since it can fold into the correct tertiary structure more rapidly than wild-type GFP. This property is important for experiments with *E. coli*, in which incorrect folding products are readily generated in the oxidative environment of the periplasm^41^. sfGFP was fused to the C-terminus of CsgA via an Ser-Asp-Phe-Met linker (Fig. 6a) that minimizes unexpected interference between two domains^42^. The gene encoding CsgA-sfGFP was cloned downstream of *csgB* in the plasmid, since it was observed that expressing CsgA-sfGFP alone resulted in aggregation whereas co-expression with CsgB did not. This fusion protein is transported to the periplasm but is not translocated across the outer membrane since folded sfGFP cannot pass through the narrow CsgG channel on the outer membrane^11,12^. We introduced the CsgA-sfGFP expression plasmid into wild-type and ∆*dnaK* cells. As expected, sfGFP fluorescence was observed at the periphery of wild-type cells, indicating that CsgA-sfGFP was translocated to the periplasm (Fig. 6b). In contrast, numerous fluorescent foci were observed in the cytoplasm of ∆*dnaK* cells. The fact that sfGFP alone did not form aggregates in the cytoplasm of either wild-type or ∆*dnaK* cells suggested that the observed aggregation was due to CsgA. Cell fractionation and fluorescence analysis also revealed that a large proportion of CsgA-sfGFP aggregated in ∆*dnaK* cells but was present in the periplasm fraction of wild-type cells (Supplementary Fig. 12a). Immunoblotting analysis revealed that the transport precursor of CsgA-sfGFP (pre-CsgA-sfGFP) accumulated as aggregates in the cytoplasm, since aggregates in ∆*dnaK* cells had lower mobility than the periplasmic CsgA-sfGFP observed in wild-type cells by sodium dodecyl sulfate polyacrylamide gel electrophoresis (SDS-PAGE) (Supplementary Fig. 12b). Minute amounts of pre-CsgA-sfGFP were also detected in the aggregate fraction of wild-type cells (Supplementary Fig. 12b), and a subset of wild-type cells harbouring CsgA-sfGFP had foci at both the periphery and within the cytoplasm (Fig. 6b), probably due to higher expression levels that can occur stochastically within a population. An in vitro translation and protein folding assay also showed that the DnaK chaperone system is involved in the maintenance of CsgA in a soluble state, whereas neither the GroEL/ES nor SecB chaperone—both of which are known to play a role in protein transport^38,43^—prevented CsgA aggregation (Supplementary Fig. 13). Given that ∆*clpB*, ∆*ibpA*, and ∆*ibpB* were able to produce curli (Fig. 1a), it is likely that preventing the aggregation and cytoplasmic retention of CsgA by DnaK—rather than re-solubilization of pre-formed aggregates by the coordinated activities of the DnaK-ClpB bichaperone system and small Hsp—is critical for the transport of CsgA across the cytoplasmic membrane. These in vitro and in vivo data suggest that DnaK is the major chaperone regulating CsgA quality by preventing aggregation or premature fibrillation of the newly synthesized polypeptide into an export-incompetent form.

We next investigated the part of CsgA that determines its aggregation in ∆*dnaK* cells by introducing various deletion and site-specific mutations into the CsgA of CsgA-sfGFP (Fig. 6a). Plasmids expressing these CsgA-sfGFP constructs were transformed into wild-type and ∆*dnaK* cells. Unexpectedly, CsgA-sfGFP variants with the N-terminal signal peptide composed of 20 amino acids (CsgA_1–20_-sfGFP and CsgA_1–42_-sfGFP) formed aggregates in the cytoplasm of ∆*dnaK* cells, although they were translocated to the periplasm in wild-type cells. In contrast, constructs lacking the signal peptide (CsgA_21–42_-sfGFP, CsgA_21–151_-sfGFP, and CsgA_43–151_-sfGFP) showed diffuse distribution in the cytoplasm of both strains (Fig. 6c). We also introduced four mutations (Q49A, N54A, Q139A, and N144A) into the CsgA sequence to generate the CsgA_slowgo_ mutant protein, which was unable to undergo self-assembly in vitro^9^. CsgA_slowgo_-sfGFP formed aggregates and was retained in the cytoplasm of ∆*dnaK* cells but was transported to the periplasm in wild-type cells (Fig. 6c). These results indicate that the N-terminal signal peptide, but not the amyloidogenic repeat R1–5, determines CsgA aggregation. In addition, DnaK may directly bind to this N-terminal peptide and protect CsgA from aggregation, thereby enabling its transport across the cytoplasmic membrane.

### DnaK binds to signal peptides of a subset of proteins

**Fig. 7:**
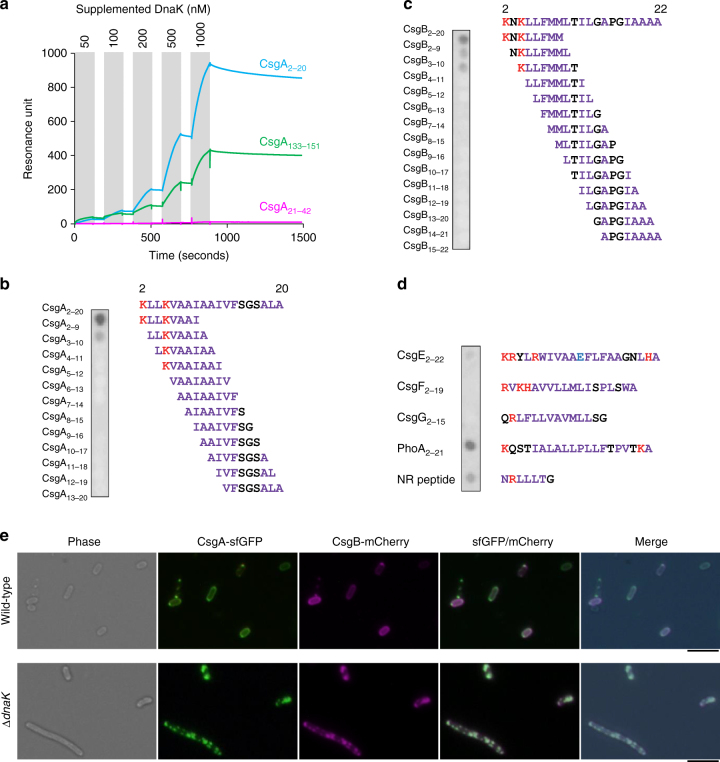
DnaK binds to the N-terminal signal peptides of CsgA and CsgB. **a**. Surface plasmon resonance single-cycle analysis was performed using CsgA peptides and indicated concentrations of DnaK as ligands and analytes, respectively. Analytes were loaded during the periods shaded in grey. **b**-**d** Peptide scanning was performed to assess DnaK recognition of the signal peptides of CsgA (**b**), CsgB (**c**), and other secreted proteins related curli production (**d**). PhoA signal peptide and NR peptide were used as positive controls. Letters in red, blue, purple, and black indicate basic, acidic, hydrophobic, and neutral amino acids, respectively. **e** CsgA-sfGFP and CsgB-mCherry were simultaneously expressed from the plasmid, pBAD-CsgB-mCherry/CsgA-sfGFP, in the wild-type and Δ*dnaK* strains. The strains were cultured in LB media supplemented with ampicillin at 30 °C overnight. Leaky expressions without any inducers were enough to visualize the respective fluorescent proteins. Phase contrast, sfGFP, mCherry, and merged images are shown

The DnaK-recognition motif consists of a hydrophobic core of four to five residues enriched in Leu, Ile, Val, Phe, and Tyr flanked by two regions containing basic residues^44^. The signal peptide of CsgA (Fig. 6a, coloured in magenta) likely contains a partial DnaK-recognition motif. We therefore investigated whether DnaK directly interacts with the signal peptide of CsgA using the three chemically synthesized peptides CsgA_2–20_ (signal peptide), CsgA_21–42_ (CsgG-recognition sequence), and CsgA_133–151_ (R5, the most amyloidogenic of the five repeats) as ligands in the surface plasmon resonance analysis. The N-terminal Met was omitted from the signal peptide, since this residue is cleaved co-translationally by ribosome-bound peptide deformylase (PDF)^45^ and methionine aminopeptidase (MAP)^46^ in many nascent polypeptides. DnaK bound strongly to CsgA_2–20_ and moderately to CsgA_133–151_, but did not bind to CsgA_21–42_ (Fig. 7a). Curve fitting for the interaction between DnaK and CsgA_2–20_ with a 1:1 binding mode yielded an association rate constant (*k*_a_) of 1.17 ± 0.228 × 10^5^ (M^−1^ s^−1^) and a dissociation rate constant (*k*_d_) of 2.56 ± 0.0268 × 10^−4^ s^−1^; *k*_a_ = 4.72 ± 0.0189 × 10^3^ (M^−1^ s^−1^) and *k*_d_ = 9.77 ± 0.130 × 10^−5^ s^−1^ were also estimated for CsgA_133–151_. These rates resulted in a dissociation constant (*K*_D_) of 2.19 × 10^−9^ M for CsgA_2–20_ and 2.07 × 10^−8^ M for CsgA_133–151_. Thus, DnaK can directly and strongly bind to the signal peptide of CsgA.

We examined the region of the signal peptide that is important for recognition by DnaK (Fig. 7b). To this end, we screened cellulose-bound peptides^44^ representing the complete sequences of the CsgA signal peptide. The peptide scans consisted of 8-amino acid peptides overlapping by seven residues and containing all potential DnaK-binding sites. Signal peptides of other proteins and known DnaK substrate peptides were also included in the scans (Fig. 7c, d). DnaK strongly bound to the N-terminal 8-amino acid peptide of CsgA (NH_2_-KLLKVAAI-COOH) (Fig. 7b) and to the corresponding peptide of CsgB (NH_2_-KNKLLFMM-COOH) (Fig. 7c). The reliability of these results was confirmed by performing the assay with known DnaK substrate peptides (PhoA_2–21_ and NR peptide)^47^ (Fig. 7d). In addition, DnaK did not bind to the signal peptides of CsgE, CsgF, or CsgG (Fig. 7d). These results indicate that DnaK can bind to the signal peptides of some proteins and may regulate the folding status and transport competence of CsgB, a minor component of curli, in addition to CsgA.

Finally, we simultaneously visualized CsgA and CsgB translocation and aggregation using CsgA-sfGFP and CsgB-mCherry fusion constructs. The fluorescent proteins were co-expressed from the plasmid pBAD-CsgB-mCherry/CsgA-sfGFP under the control of the arabinose promoter. In this experiment, arabinose supplementation was not required since there was leaky expression of both proteins that was sufficient for visualization, and addition of excess arabinose induced protein aggregation. As expected, CsgA-sfGFP and CsgB-mCherry were both translocated to the periplasm in wild-type cells but formed aggregates in the cytoplasm of ∆*dnaK* cells (Fig. 7e). These results demonstrate that DnaK is required for quality control of CsgA as well as CsgB, likely through interaction with aggregation-related signal peptides.

## Discussion

**Fig. 8:**
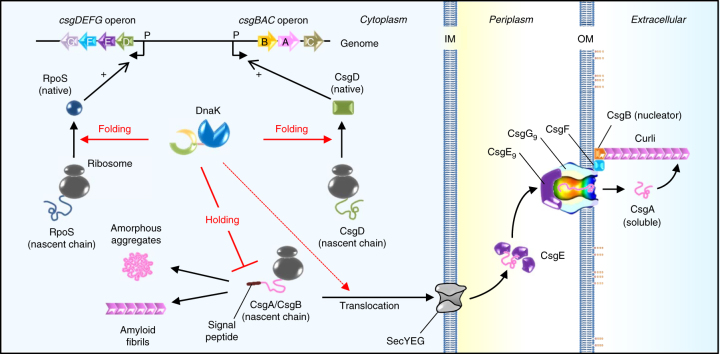
Model for multitasks of DnaK in curli biogenesis. DnaK regulates quantity and quality of RpoS, bearing expression of the *csgDEFG* operon. DnaK also assists de novo folding of CsgD, which leads to the activation of the *csgBAC* operon. DnaK recognizes N-terminal end of signal peptide of CsgA and CsgB and maintains their transport competent state by preventing aggregation, which likely accelerates successful translocation of these amyloidogenic proteins into the periplasm. Solid red lines: strongly supported by the results in this study. A dotted red line: suggested by the data

The results of this study demonstrate that DnaK has multiple roles in the biogenesis of curli, the functional extracellular amyloid fibrils that constitute biofilm (Fig. 8). DnaK regulates the quantity and quality of RpoS to ensure expression of the *csg* genes responsible for curli production. DnaK is also required for de novo folding of the master transcriptional regulator CsgD, which leads to the expression of the curli structural components CsgA and CsgB. Additionally, it contributes to the maintenance of CsgA and CsgB in a transport-competent state by directly binding to the aggregation-prone N-terminal signal peptides, which is indispensable for translocation of these amyloidogenic proteins to the periplasm.

RpoS is mainly degraded by ClpXP, an ATP-dependent protease. On the other hand, overproduction of CsgD can affect the cellular protein level of RpoS via the IraP protein in a feed-forward loop^48^. IraP is known to antagonize RssB, an adaptor protein for the ClpXP protease that degrades RpoS^49^. Overproduction of CsgD enhanced the transcription of *iraP*, leading to accumulation of RpoS^48^. In the present study, the soluble RpoS level in ∆*csgD* is comparable to that in wild type (Fig. 3a and Supplementary Fig. 14), suggesting that the effect of CsgD on the cellular RpoS level is observed only when CsgD is overproduced. Of note, CsgD does not affect the solubility of RpoS. Importantly, our data indicate that DnaK positively regulates the amount of RpoS (Fig. 3a) through its canonical Hsp70 chaperone function (Fig. 3b). DnaK may directly or indirectly protect RpoS from degradation by ClpXP^33^. However, the molecular basis for the quantity control of RpoS by DnaK and the requirement for the co-chaperones DnaJ and GrpE in this activity remain unclear. Additionally, although it is thought that DnaK does not function alone and always acts in concert with co-chaperones in the regulation of proteostasis, it is not known whether this cooperativity is required for certain cellular processes.

An outstanding question concerns the mechanism by which DnaK controls the quality of other regulators. Transcriptional regulation of curli biogenesis is a complex process involving many genes^29^ that may be directly or indirectly mediated by DnaK. Alternatively, DnaK may be involved in the post-transcriptional regulation and stabilization of gene products.

Our genetic screen of protein quality control systems involved in biofilm formation demonstrated that Lon and ClpXP proteases may be involved in this process in addition to DnaK, whereas other known non-essential cytoplasmic chaperones and proteases, periplasmic chaperones, and membrane proteases were dispensable (Fig. 1a and Supplementary Fig. 1). Clearance of misfolded and/or aggregated proteins by these proteases may be important for biofilm formation. Our analyses did not include SecA and GroEL/ES—two molecular chaperones that participate in protein transport^50^ and folding^51,52^—due to the lethality associated with their deficiency. A global analysis of chaperone effects on *E. coli* protein folding demonstrated that GroEL/ES induced CsgD solubility in vitro^37^, suggesting that chaperonin contributes to curli biogenesis. However, given that single deletion of the *dnaK* gene severely attenuated curli production (Fig. 1a) and that DnaK rather than GroEL/ES prevented CsgA aggregation in vitro (Supplementary Fig. 13), DnaK is likely the primary chaperone in curli production.

DnaK targets the signal peptides of CsgA and CsgB co- or post-translationally during their translocation, thereby maintaining their transport-competent states. Trigger factor encoded by *tig* is a ribosome-associated molecular chaperone that co-translationally assists in the folding of nascent peptide chains^53^ but is dispensable for protein export and stability^54^. Indeed, *tig* deletion did not affect biofilm formation or curli production (Fig. 1a). SecB is a non-essential, ATP-independent holdase^55^ that participates in the export of just 4% of the *E. coli* secretome^50^. SecB substrates include CsgF, an accessory protein in curli biogenesis^56^. Although the ∆*csgF* strain did not produce curli (Supplementary Fig. 7), *secB* deletion did not affect curli production (Fig. 1a) and SecB was unable to maintain CsgA in a soluble form in vitro (Supplementary Fig. 13). These results indicate that DnaK, but neither trigger factor nor SecB, modulates the export of amyloid-forming proteins.

It was striking that CsgA_21–151_-sfGFP showed a dispersed fluorescence in the cytoplasm regardless of the presence or absence of DnaK, suggesting it does not assemble into curli fibres under the tested conditions. Previously, it was reported that cytoplasmic molecular chaperone Hsp33, in addition to DnaK, can prevent aggregation of CsgA_21–151_ in vitro^23^. Therefore, there might be a redundant chaperone (e.g., Hsp33 and others) for premature CsgA fibrillation in the cytoplasm.

We used fluorescent protein fusions to visualize CsgA and CsgB export and folding states. Recently, the putative periplasmic chaperones CsgC, CsgE, and Spy were shown to inhibit CsgA amyloid formation in vitro^14,16,23^. Our imaging system can be used to evaluate in vivo functions of these proteins. Based on the observation that single knockouts of *csgC* and *spy* did not affect curli production (Fig. 1a and Supplementary Fig. 1), it is likely that CsgE alone or in conjunction with CsgC and Spy modulates quality control of CsgA and CsgB in the periplasm. Alternatively, periplasmic proteases may mediate proteostasis of these amyloidogenic proteins. These possibilities are currently under investigation.

In bacteria, the exit channel of the large ribosomal subunit can accommodate an extended peptide of ∼30 amino acids^57^. Shortly after peptide exit, the formyl group of the N-terminal formylmethionine is processed by PDF^45^ and MAP removes the remaining methionine^46^. Given that the N-terminal 8-amino acids of CsgA and CsgB were recognized by DnaK (Fig. 7b, c) and that ribosome-associated Hsp70 functions co-translationally in eukaryotic protein homoeostasis^58^, DnaK may also act co-translationally on a subset of nascent peptides and maintain them in a transport-competent state. Moreover, other aggregation-prone signal peptides may be present in the secretome; a genome-wide analysis of the contribution of DnaK to protein transport can clarify this point.

We recently showed that myricetin, a flavonol produced by plants, inhibits the cellular functions of DnaK and thereby blocks curli-dependent biofilm formation in *E. coli*^24^. In addition, myricetin treatment sensitized *E. coli* to vancomycin, an aminoglycoside antibiotic that has a less potent antimicrobial effect on Gram-negative bacteria^24^. Myricetin also inhibited biofilm formation by *S. aureus*, including clinically isolated methicillin-resistant strains^24^. The results of the present study are not only important for understanding the basic principle of proteostasis regulated by molecular chaperones, but can also serve as a basis for the development of a new class of anti-biofilm therapeutics. Given that DnaK is a member of the highly conserved Hsp70, our findings also provide insight into amyloid biology and neurodegenerative diseases.

## Methods

### Bacterial strains and culture conditions

*E. coli* strains used in this study are listed in Supplementary Table 1. All strains were cultured in LB medium or YESCA (1% casamino acid, 0.1% yeast extracts) medium. When required, the medium was supplemented with 30 μg mL^−1^ chloramphenicol or 100 μg mL^−1^ ampicillin.

### Plasmid construction

Plasmids expressing DnaK mutants (DnaK_K70A_ and DnaK_V436F_) were generated by inverse PCR site-directed mutagenesis using Phusion High-Fidelity DNA polymerase (New England Biolabs, Tokyo, Japan), pDnaK_WT_ as a template, and the primer sets dnaK-K70A-F/dnaK-K70A-R and dnaK-V436F-F/dnaK-V436F-R, respectively. The resultant plasmids were named pDnaK_K70A_ and pDnaK_V436F_, respectively.

The empty plasmid pCA24N was linearized by inverse PCR using Phusion High-Fidelity DNA polymerase and primers pCA24N-Art-F and pCA24N-Art-R. The *rpoS* gene was amplified by PCR from the *E. coli* JM109 genome using Phusion High-Fidelity DNA polymerase and primers rpoS-Art-F and rpoS-Art-R. The *rpoS-mcherry* gene was amplified by PCR from MG1655 *rpoS::mcherry* genomic DNA^42,59^ using KOD Plus DNA polymerase ver. 2 (Toyobo, Osaka, Japan) and primers rpoS-Art-F and rpoS-mcherry-R. The *csgD* gene was amplified by PCR from the *E. coli* JM109 genome using Phusion High-Fidelity DNA polymerase and primers csgD-Art-F and csgD-Art-R. A DNA fragment encoding CsgDEFG was amplified by PCR from the *E. coli* JM109 genome using Phusion High-Fidelity DNA polymerase and primers csgD-Art-F and csgG-Art-R. The DNA fragment encoding C-terminally His_5_-tagged CsgA was amplified by PCR from the *E. coli* JM109 genome using Phusion High-Fidelity DNA polymerase and primers csgA-Art-F and csgA-His5-Art-R. These DNA fragments were cloned into linearized pCA24N using a GeneArt seamless cloning kit (Thermo Fisher Scientific, Waltham, MA, USA). For construction of pCA24-mCherry, inverse PCR was performed using KOD Plus Neo DNA polymerase (Toyobo), primers mcherry-inv-F and mcherry-inv-R, and pRpoS-mCherry as a template.

DNA fragments encoding CsgBA and CsgEFG were amplified separately by PCR from the *E. coli* JM109 genome using Phusion High-Fidelity DNA polymerase and the following primer sets csgB-Art-F/csgA-Art-R and csgE-Art-F/csgG-Art-R, and cloned into linearized pCA24N using the GeneArt seamless cloning kit. The resulting plasmid was named pCsgBAEFG.

To construct pCsgD*EFG, a helix-turn-helix motif coding region within *csgD* was deleted from pCsgDEFG by inverse PCR using KOD Plus DNA polymerase (Toyobo) and primers csgD*-F and csgD*-R.

The DNA fragment containing the p15A origin and *cat* gene encoding chloramphenicol acetyltransferase was amplified by PCR from pBAD33 using Phusion High-Fidelity DNA polymerase and primers pBAD33-3000-Art-F and pBAD33-1-R. The DNA fragment containing *araC*, the P_BAD_ promoter, the multi-cloning site, and the ampicillin-resistance cassette was amplified by PCR from pBAD/Myc-His B using Phusion High-Fidelity DNA polymerase and primers pBAD/Myc-His-3033-F and pBAD/Myc-His-1959-R. The DNA fragments were ligated using the GeneArt seamless cloning kit; the resultant plasmid was named pBAD/SS01. The chloramphenicol-resistance cassette was removed from pBAD/SS01 by inverse PCR using KOD plus Neo DNA polymerase and primers pBAD33-delta-CP-F and pBAD33-delta-CP-R. The amplified fragment was self-ligated and named pBAD/SS02. For gene cloning, pBAD/SS02 was linearized by PCR using Phusion High-Fidelity DNA polymerase and primers pBAD-mcs-F and pBAD-mcs-R.

The DNA fragment encoding sfGFP was amplified by PCR from psfGFP using KOD plus Neo DNA polymerase and primers sfGFP-Art-F and sfGFP-Art-R, and then cloned into pBAD/SS02 using the GeneArt seamless cloning kit; the resultant plasmid was named pBAD-sfGFP.

For gene cloning, pBAD-sfGFP was linearized by inverse PCR using Phusion High-Fidelity DNA polymerase and primers pBAD-sfGFP-inverse-F and pBAD-mcs-R. The gene encoding CsgBA from *E. coli* JM109 was amplified by PCR using KOD plus Neo DNA polymerase as well as primers pBAD-csgB-Art-F and pBAD-csgA-Art-R, which generated a Ser-Asp-Phe-Met peptide linker between the C terminus of CsgA and N terminus of sfGFP. The fragments were ligated using the GeneArt seamless cloning kit and the resultant plasmid was named pBAD-CsgBA-sfGFP.

To introduce mutations in *csgA*, inverse PCR was performed using KOD plus Neo DNA polymerase, pBAD-CsgBA-sfGFP or pBAD-CsgBA_1–42_-sfGFP as a template, and the following primer sets csgA1-42-sfGFP-F/csgA1-42-sfGFP-R, csgA1-20-sfGFP-F/csgA1-20-sfGFP-R, csgA43-151-sfGFP-F/csgA43-151-sfGFP-R, csgA21-151-sfGFP-F/csgA21-151-sfGFP-R, Q49A_N54A-F/Q49A_N54A-R, and Q139A_N144A-F/Q139A_N144A-R. The resultant mutant plasmids were named pCsgBA_1–42_-sfGFP, pCsgBA_1–20_-sfGFP, pCsgBA_43–151_-sfGFP, pCsgBA_21–151_-sfGFP, pCsgBA_21–42_-sfGFP, and pCsgBA_slowgo_-sfGFP.

To construct pBAD-CsgB-mCherry/CsgA-sfGFP, pBAD-CsgBA-sfGFP was linearized by inverse PCR using KOD Plus Neo DNA polymerase and primers csgB-csgA-inter-F and csgB-csgA-inter-R. The DNA encoding mCherry was amplified by PCR from the MG1655 *rpoS::mcherry* genome using KOD Plus Neo DNA polymerase and primers csgB-mcherry-Art-F and csgB-mcherry-Art-R. The fragments were ligated using the GeneArt seamless cloning kit.

The *secB* gene from JM109 was amplified by PCR using Phusion High-Fidelity DNA polymerase and primers secB-Art-F and secB-Art-R and then cloned into pCold I using the GeneArt seamless cloning kit. The resultant plasmid was named pCold-SecB.

DNA sequences of the constructed plasmids were verified by sequence analysis (Eurofins Genomics, Tokyo, Japan). Oligonucleotide primers used in this study were synthesized by Thermo Fisher Scientific and are summarized in Supplementary Table 2.

### Biofilm formation

All strains were grown in LB medium at 30 °C overnight with shaking at 150 rpm. The cultures were diluted 1000-fold in 200 μL fresh YESCA medium (0.1% yeast extract, 1% casamino acids) and grown for the indicated periods at 30 °C in 96-well flat-bottom polystyrene plates (Corning Inc, Corning, NY, USA) to induce biofilm formation. If necessary, media were supplemented with ampicillin (100 μg mL^−1^) or chloramphenicol (30 μg mL^−1^). After removal of planktonic cells and media, biofilms were stained with 0.2% crystal violet, extracted with 99.5% ethanol, and quantified by measuring the absorbance of the extracted dye at 595 nm on a microtiter plate reader (Infinite F200 Pro; Tecan, Männedorf, Switzerland).

### CR-binding assay

Cells were cultured overnight in LB medium at 30 °C with shaking at 150 rpm. Aliquots (5 μL) of the cultures were spotted on YESCA plates (0.1% yeast extract, 1% casamino acids, 2% agar) supplemented with 10 μg mL^−1^ CR and 10 μg mL^−1^ Coomassie Brilliant Blue (CBB) G250 that were incubated at 30 °C for 48–72 h to induce curli production. When required, plates were supplemented with ampicillin (100 μg mL^−1^) or chloramphenicol (30 μg mL^−1^).

### Protein purification

N-terminal His-tagged DnaK (His-DnaK) was overexpressed from pDnaK_WT_ (ASKA clone^60^) in *E. coli* BL21 (DE3) cells, which were grown at 30 °C in LB medium containing 30 μg mL^−1^ chloramphenicol; His-DnaK expression was induced by adding 0.1 mM isopropyl-1-thio-β-d-galactopyranoside (IPTG), followed by incubation at 30 °C for 3 h. Cells from 1-L culture were harvested by centrifugation and resuspended in 50 mL of buffer A [20 mM Tris-HCl (pH 8.0) and 300 mM NaCl] supplemented with protease inhibitor cocktail (Nacalai Tesque, Kyoto, Japan). After sonication on ice, cell lysates were centrifuged at 9000 × *g* for 30 min at 4 °C, and the supernatant was loaded onto a 2-mL bed volume of TALON resin (Clontech, Palo Alto, CA, USA) that was washed with buffer A supplemented with 5 mM imidazole. Recombinant proteins were eluted using 250 mM imidazole and purified using a Mono Q column (GE Healthcare, Pittsburgh, PA, USA) and a 0–1000 mM NaCl gradient in buffer B [20 mM Tris-HCl (pH 8.0), 1 mM DTT, and 10% (w/v) glycerol]. Each fraction containing His-DnaK was separately stored and further purified by size exclusion chromatography (Superdex G-200, GE Healthcare) in buffer C [20 mM Tris-HCl (pH 8.0), 1 mM DTT, 10% (w/v) glycerol, and 100 mM NaCl]. Purified His-DnaK was pooled and quantified using a Bradford Assay Kit (Bio-Rad Laboratories, Hercules, CA, USA).

Purification procedures for other proteins are described in Supplementary methods.

### Peptides

To generate polyclonal antibodies, peptides CsgA-PEP (NH_2_-CLDQWNGKNSEMTVKQFGGGN-COOH), CsgB-PEP (NH_2_-CEGSSNRAKIDQTGDY-COOH), and CsgG-PEP (NH_2_-CDGIDRGLWDLQNKAERQ-COOH) were generated by Medical Biological Laboratories (MBL) Co. (Aichi, Japan) according to previous reports^18,61^. The N-terminal cysteine was used for conjugation of the carrier (KLH) and for affinity purification. These peptides were purified (>90%) by HPLC.

For surface plasmon resonance analysis, CsgA_2–20_ (NH_2_-KLLKVAAIAAIVFSGSALA-COOH), CsgA_21–42_ (NH_2_-GVVPQYGGGGNHGGGGNNSGPN-COOH), CsgA_133–151_ (NH_2_-SSVNVTQVGFGNNATAHQY-COOH) were synthesized and purified (>95%) by HPLC by Eurofins Genomics.

### Antibodies

Rabbit anti-CsgA, -CsgB, and -CsgG antisera were developed and purified using antigen-conjugated affinity resins. Rabbit anti-CsgD-Myc-His and rabbit anti-FtsZ were developed by Eurofins Genomics. The anti-CsgD-Myc-His antibody was further purified with CsgD-Myc-His conjugated affinity resin. Rabbit anti-polyribonucleotide phosphorylase (PNPase) was developed by Scrum (Tokyo, Japan). Mouse monoclonal anti-RpoS, -DnaK, and -Maltose-binding protein (MBP) antibodies were purchased from Abcam (Cambridge, MA, USA), Stressgen Bioreagents (Ann Arbor, MI, USA), and Thermo Fisher Scientific, respectively. Rabbit anti-CsgD and -OmpA were provided by Drs. A. Ishihama and Y. Akiyama, respectively. Horseradish peroxidase (HRP)-conjugated goat anti-rabbit and anti-mouse IgG (Bio-Rad Laboratories) secondary antibodies were also used in this study. Anti-His HRP conjugate (Qiagen, Hilden, Germany) was used for peptide scanning as described below.

### Microarray and real-time-PCR

Overnight cultures were grown in LB medium at 30 °C with shaking. Aliquots (5 μL) of the cultures were diluted in 5 mL fresh YESCA medium in six-well plates and incubated for 48 h at 30 °C. Total RNA was purified using the RNeasy Mini Kit (Qiagen) according to the manufacturer’s instruction. Isolated RNA was used for microarray analysis using the Agilent *E. coli* Gene Expression Microarray (Takara, Otsu, Japan). Relative expression levels of transcripts in the *dnaK* null mutant were compared with wild-type levels. Average log_2_ fold-change values with standard errors were calculated from four comparative analyses.

The transcript levels of *csgA* and *csgD* were measured by real-time-PCR using primer sets RT-csgA-F/RT-csgA-R and RT-csgD-F/RT-csgD-R, respectively. cDNA was generated using the Prime Script II 1st strand cDNA Synthesis Kit (Takara) according to the manufacturer’s instruction. Real time-PCR reactions were performed on a Real Time PCR 7500 Fast system (Applied Biosystems, Foster City, CA, USA).

### In vivo protein folding assay

Overnight cultures were grown in LB medium at 30 °C with shaking at 150 rpm. Aliquots (30 μL) of the cultures were diluted to 30 mL YESCA medium and incubated for 48 h at 30 °C under static conditions. After centrifugation at 5000 × *g* for 10 min at 4 °C, bacterial pellets were resuspended in ice-cold STE buffer [10 mM Tris-HCl (pH 8.0), 100 mM NaCl, 2 mM EDTA]. The cells were disrupted by sonication (five times for 20 s each) on ice and centrifuged at 20,000 × *g* for 10 min at 4 °C to obtain soluble fractions. Protein concentrations of the soluble fractions were measured with the Bradford Assay Kit. Ten micrograms of soluble fraction and an equivalent volume of total fraction (before centrifugation) were separated by SDS-PAGE on SDS-15% polyacrylamide gels, which were stained with CBB. To detect RpoS and CsgD, immunoblotting was performed as described below.

### In vitro protein synthesis and folding assay

Cell-free synthesis of CsgD was performed using the PURE system composed of purified recombinant elements^36^. The *csgD* gene was amplified by PCR from the CsgD-expression plasmid pASKA-CsgD using KOD plus DNA polymerase ver. 2 and the primer set Pure-Niwa-F^37,62^ and Pure-CsgD-R. The resultant DNA fragment was incubated at 37 °C for 4 h with recombinant protein synthesis solution (PURE*frex*; GeneFrontier, Kashiwa, Japan). When required, the reaction was supplemented with purified DnaK (5 μM), DnaJ (1 μM), and GrpE (1 μM). After incubation, small aliquots of the solution were collected as the total fraction and the residue was centrifuged at 20,000 × *g* for 10 min at 4 °C. Equivalent volumes of the total, soluble, and insoluble fractions were mixed with 2× SDS sample buffer [150 mM Tris-HCl (pH 6.8), 4% SDS, 20% glycerol, 10% 2-mercaptoethanol] and resolved on SDS-15% polyacrylamide gels that were stained with CBB. CsgD was detected by immunoblotting as described below.

The *csgA* gene was also amplified by PCR from the CsgA-expression plasmid pCsgA-His using KOD plus DNA polymerase ver. 2 and the primer set Pure-Niwa-F^37,62^ and csgA-His5-Art-R. The resultant DNA fragment was incubated at 37 °C for 3 h with the PURE*frex* solution. When required, the reaction was supplemented with DnaK Mix containing DnaK (5 μM), DnaJ (1 μM), and GrpE (1 μM) (GeneFrontier), GroE Mix composed of GroEL_14-mer_ (1 μM) and GroES_7-mer_ (2 μM) (GeneFrontier), and purified SecB. After incubation, small aliquots of the solution were obtained as the total fraction and the residue was centrifuged at 20,000 × *g* for 10 min at 4 °C. Equivalent volumes of the total, soluble, and insoluble fractions were resolved on SDS-15% polyacrylamide gels that were stained with CBB. CsgA was detected by immunoblotting as described below. GroEL Mix slightly inhibited translation of CsgA, as noted by the manufacturer.

### Gel-shift assay

The DNA-binding activity of CsgD in the cell-free transcription/translation system was analysed with gel-shift assay. To construct the probe, the *csgB* promoter was amplified by PCR using the Alexa 488-labelled forward primer csgB-UTR-Alexa-F and the label-free reverse primer csgB-UTR-R; the fragment was purified using the QIAquick PCR Purification Kit (QIAGEN). The labelled probe (5 pg) was incubated in gel-shift assay buffer [10 mM Tris-HCl (pH 7.5), 150 mM NaCl, 3 mM MgCl_2_, 5% glycerol] without supplementation or supplemented with cell-free transcription/translation solution containing in vitro-synthesized CsgD. Non-specific DNA (100 μg mL^−1^ salmon sperm DNA; Wako Pure Chemical Industries, Osaka, Japan) and bovine serum albumin (BSA; 100 μg mL^−1^) (Thermo Fisher Scientific) were added to block non-specific binding. When required, cell-free transcription/translation solution without either the *csgD* gene or the DnaK (5 μM)/DnaJ (1 μM)/GrpE (1 μM) mixture were used as negative controls. After the binding reaction at 25 °C for 20 min, samples were resolved by electrophoresis on a 7.5% SuperSep Ace polyacrylamide gel (Wako Pure Chemical Industries) under native conditions at 25 °C in 1:2 Tris-bornate-EDTA buffer. Fluorescence signal from the probe was detected with an LAS-4000 Image Analyzer (GE Healthcare).

### Cell fractionation

*E. coli* cells grown in YESCA medium supplemented with 30 μg mL^−1^ chloramphenicol at 30 °C for 48 h were centrifuged at 5000 × *g* for 10 min at 4 °C. The pellet was resuspended in spheroplast buffer [10 mM Tris-HCl (pH 8.0), 30% sucrose, 5 mM EDTA, and 1 mg mL^−1^ lysozyme] and incubated on ice for 45 min. After centrifugation at 26,000 × *g* for 10 min at 4 °C, periplasmic and spheroplast fractions were collected as the supernatant and pellet, respectively. Spheroplasts were resuspended in cytoplasmic buffer [10 mM Tris-HCl (pH 8.0) and 30% sucrose] and disrupted by sonication (five times for 20 s each) on ice. Soluble and insoluble fractions were separated by centrifugation at 20,000 × *g* for 10 min at 4 °C. The insoluble fraction was resuspended in the same volume of cytoplasmic buffer and used as the aggregate fraction. The soluble fraction was ultracentrifuged at 100,000 × *g* for 10 min at 4 °C to separate the soluble cytoplasmic fraction and insoluble membrane fraction. The latter was dissolved in cytoplasmic buffer. Equivalent volumes of the membrane, periplasm, cytoplasm, and aggregates fractions were separated by SDS-PAGE on SDS-15% polyacrylamide gels that were stained with CBB. CsgA and other fraction marker proteins were detected by immunoblotting as described below.

### Interbacterial complementation assay

*E. coli* ∆*csgA* was used as an acceptor for an interbacterial complementation assay, since the surface-localized CsgB in this strain can serve as an acceptor of secreted CsgA. *E. coli* ∆*csgB* was used as a donor of secreted CsgA. Overnight cultures of the indicated *E. coli* strains were mixed with the equivalent volume of ∆*csgA* or ∆*csgB*, and aliquots (2.5 μL) of mixtures were spotted on YESCA plates containing 10 μg mL^−1^ CR and 10 μg mL^−1^ CBB. As controls, individual cultures were also spotted with neither the acceptor nor donor strain. Plates were incubated at 30 °C for 48–72 h.

### Immunoblotting

After SDS-PAGE, proteins were transferred to polyvinylidene difluoride membranes using the iBlot 2 dry blotting system (Thermo Fisher Scientific) according to the manufacturer’s instructions. Membranes were treated with blocking solution composed of 1–5% skimmed milk in Tris-buffered saline containing 0.1% (v/v) Tween 20 (TBS-T) for at least 1 h or overnight at 25 °C. After gentle washing with TBS-T, the membrane was probed with antibodies against CsgA (1:200), CsgB (1:200), CsgD (1:5000), CsgG (1:200), RpoS (1:2000), DnaK (1:5000), FtsZ (1:5000), OmpA (1:5000), PNPase (1:5000), or MBP (1:5000) diluted in CanGet Signal 1 (Toyobo) for at least 1 h or overnight at 25 °C. Membranes were washed twice with TBS-T. To detect CsgA, CsgB, CsgD, CsgG, FtsZ, PNPase, and OmpA, membranes were then incubated with HRP-conjugated goat anti-rabbit IgG antibody (1:50,000 in CanGet Signal 2; Toyobo) for 1 h at 25 °C. To detect RpoS, DnaK, and MBP, membranes were incubated with HRP-conjugated goat anti-mouse IgG antibody (1:2000 in CanGet Signal 2) for 1 h at 25 °C. After three washes with TBS-T, signals were detected using the ECL Prime Western Blotting Detection Reagent (GE Healthcare) and LAS-4000 Image Analyzer. If required, signals were quantified with ImageQuant TL software ver. 7.0 (GE Healthcare).

To detect CsgA and CsgB monomers, curli fibres were depolymerized into subunits by treatment with hexafluoroisopropanol prior to SDS-PAGE. Bacterial cells (1 mg) were resuspended in 10 μL STE buffer and mixed with 100 μL hexafluoroisopropanol. After sonication in a water bath for 10 min at room temperature, samples were vacuum dried with a SpeedVac vacuum concentrator (Thermo Fisher Scientific) at 45 °C for more than 30 min. Dried samples were dissolved in 20 μL of 8 M urea solution and sonicated in water bath for 5 min at room temperature. Solutions were mixed with equal volume of 2× SDS sample buffer and aliquots (5 μL) were separated by SDS-PAGE on SDS-15% polyacrylamide gels.

### Transmission electron microscopy

To visualize curli produced in the extracellular milieu, *E. coli* strains were grown on YESCA plates at 30 °C for 3 days. Colony biofilm cells were resuspended in phosphate-buffered saline, applied to a carbon-coated copper grid, and stained with 2% uranyl acetate. Samples were analysed using a transmission electron microscope (JEM-1400; JEOL, Tokyo, Japan) at a voltage of 80 kV.

### Fluorescence microscopy

*E. coli* cells expressing RpoS-mCherry were grown in LB medium supplemented with 30 μg mL^−1^ chloramphenicol overnight at 30 °C. The cultures were diluted 1000-fold in YESCA medium supplemented with 30 μg mL^−1^ chloramphenicol and incubated at 30 °C for 72–96 h. Leaky expression from the *lac* promoter in the absence of IPTG was sufficient for protein visualization.

*E. coli* cells expressing CsgA-sfGFP, CsgA_1–20_-sfGFP, CsgA_21–42_-sfGFP, CsgA_1–42_-sfGFP, CsgA_21–151_-sfGFP, CsgA_43–151_-sfGFP, CsgA_slowgo_-sfGFP, or sfGFP were grown overnight at 30 °C in LB medium supplemented with 100 μg mL^−1^ ampicillin. Cultures were diluted 1000-fold in YESCA medium supplemented with 100 μg mL^−1^ ampicillin and incubated at 30 °C for 24 h. The arabinose concentrations were as follows: 0.0002% (CsgA-sfGFP, CsgA_1–42_-sfGFP, sfGFP), 0.002% (CsgA_1–20_-sfGFP, CsgA_slowgo_-sfGFP), 0.02% (CsgA_21–42_-sfGFP, CsgA_21–151_-sfGFP, CsgA_43–151_-sfGFP). Lower concentrations of arabinose were used to induce aggregation-prone proteins.

*E. coli* cells coexpressing CsgA-sfGFP and CsgB-mCherry were grown overnight at 30 °C in LB medium supplemented with 100 μg mL^−1^ ampicillin. Leaky expression from the arabinose pBAD promoter was sufficient for protein visualization.

The fluorescence signal from sfGFP and mCherry in these cultures was visualized with a fluorescence microscope (Nikon, Tokyo, Japan) equipped with B2 (excitation filter, 450–490 nm; barrier filter, 520 nm) and G2A (excitation filter, 510–560 nm; barrier filter, 590 nm) filters.

### Surface plasmon resonance analysis

Surface plasmon resonance analysis of DnaK binding to CsgA peptides was performed using a BIAcore T200 system (GE Healthcare) at 25 °C in HBS-DM buffer containing 10 mM HEPES (pH 7.4), 150 mM NaCl, 5 mM MgCl_2_, and 1% dimethylsulfoxide at a flow rate of 30 μL min^−1^. The CsgA peptides CsgA_2–20_, CsgA_21–42_, and CsgA_2–20_ were immobilized on three of four flow cells of a research-grade CM7 sensor chip via *N*-ethyl-*N′*-(dimethylaminopropyl) carbodiimide *N*-hydroxy-succinimide crosslinking according to the manufacturer’s protocol. The remaining flow cell was used as a negative control. DnaK at concentrations ranging from 50–1000 nM in HBS-DM buffer was injected according to the single-cycle kinetics method. Kinetic parameters—i.e. dissociation constant (*K*_D_), binding rate (*k*_a_), and dissociation rate (*k*_d_)—were calculated by curve fitting according to the manufacturer’s instructions.

### Peptide scanning assay

A CelluSpot peptide array derived from CsgA and CsgB spotted onto glass slides was synthesized by Intavis Bioanalytical Instruments AG (Tübingen, Germany)^63^. The peptide array also contained the signal peptides of CsgE, CsgF, CsgG, and PhoA as well as NR peptide (NH_2_-NRLLLTG-COOH), a model DnaK substrate^47^. For quality control, each glass slide contained two copies of the array.

The peptide array was immersed for 30 min in blocking solution (5% BSA in TBS-T) and prewashed in DnaK binding (DB) buffer [25 mM Tris-HCl (pH 7.5), 150 mM NaCl, 10 mM KCl, 0.05% Tween 20, and 5% sucrose]. The array was incubated overnight at 4 °C with His-DnaK diluted in DB buffer to a final concentration of 50 nM. After two washes with TBS-T for 5 min at 25 °C, the array was incubated with HRP-conjugated anti-His antibody (1:10,000 in blocking buffer) for 3 h at 25 °C. The array was washed three times with TBS-T for 5 min at 25 °C. The binding of DnaK to peptides was detected with ECL Prime Western Blotting Detection Reagent and LAS-4000 Image Analyzer.

### Thermotolerance assay

Thermotolerance of the various strains was evaluated as previously described^64,65^. Briefly, overnight cultures were serially diluted 10-fold in fresh LB medium, and 5 μL of these dilutions were spotted onto LB agar plates supplemented with 30 μg mL^−1^ chloramphenicol that were incubated at 30 °C or 42 °C for 24 h.

### Statistical analysis

The two tailed Student’s *t-*test was used to assess RpoS-mCherry foci formation in *E. coli* cells and solubility of CsgD synthesized by the cell-free translation PURE System using Microsoft Excel software. For all analyses, a *P*-value of <0.05 was considered statistically significant.

### Data availability

Microarray data have been deposited in the GEO under accession number GSE102347.

## Electronic supplementary material

Supplementary Information

Description of Additional Supplementary Files

Supplementary Data 1
