# Supplementary material for: Multitasking of Hsp70 chaperone in the biogenesis of bacterial functional amyloids
Source: Commun Biol. 2018 May 31;1:52. doi: 10.1038/s42003-018-0056-0 (PMC6123696; doi:10.1038/s42003-018-0056-0)
Supplement: Supplementary file 1 — Supplementary Information [file 42003_2018_56_MOESM1_ESM.pdf]

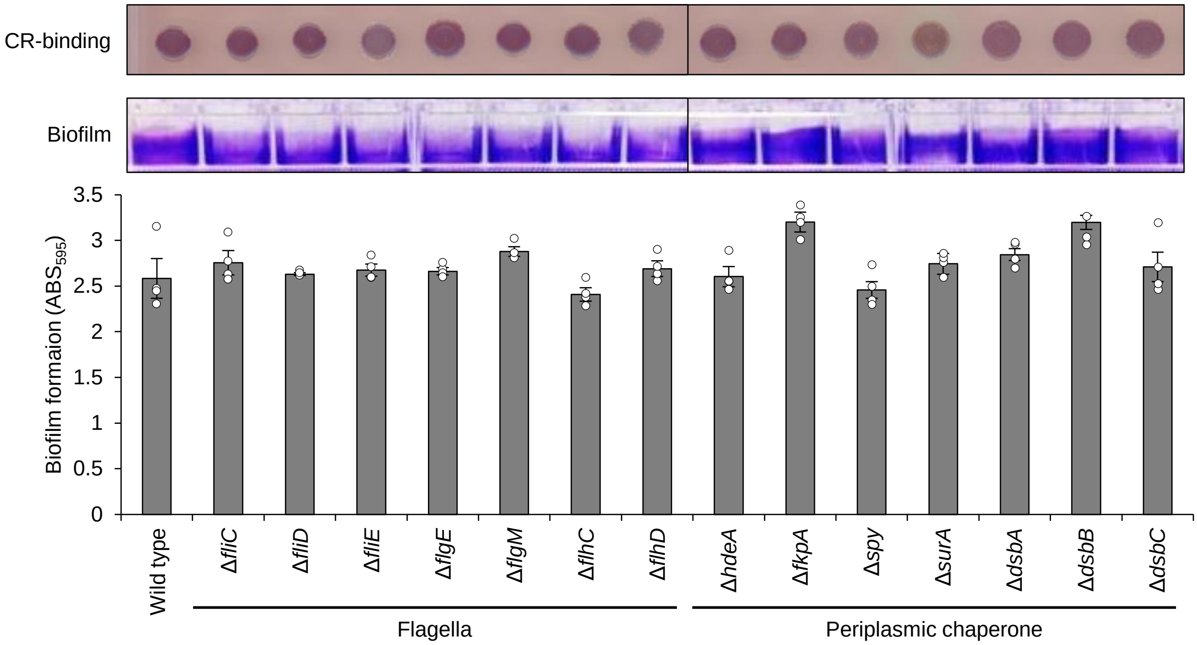

Supplementary Figure 1 Biofilm formation and curli production by various *E. coli* mutants. Curli production and biofilm formation of BW25113 wild type and the indicated isogenic mutants lacking genes responsible for flagella production and protein quality control in periplasm were analysed by Congo Red (CR)-binding assay and crystal violet staining as shown in Figure 1. Experiments were repeated at least three times. Means and standard errors and data plots are shown.

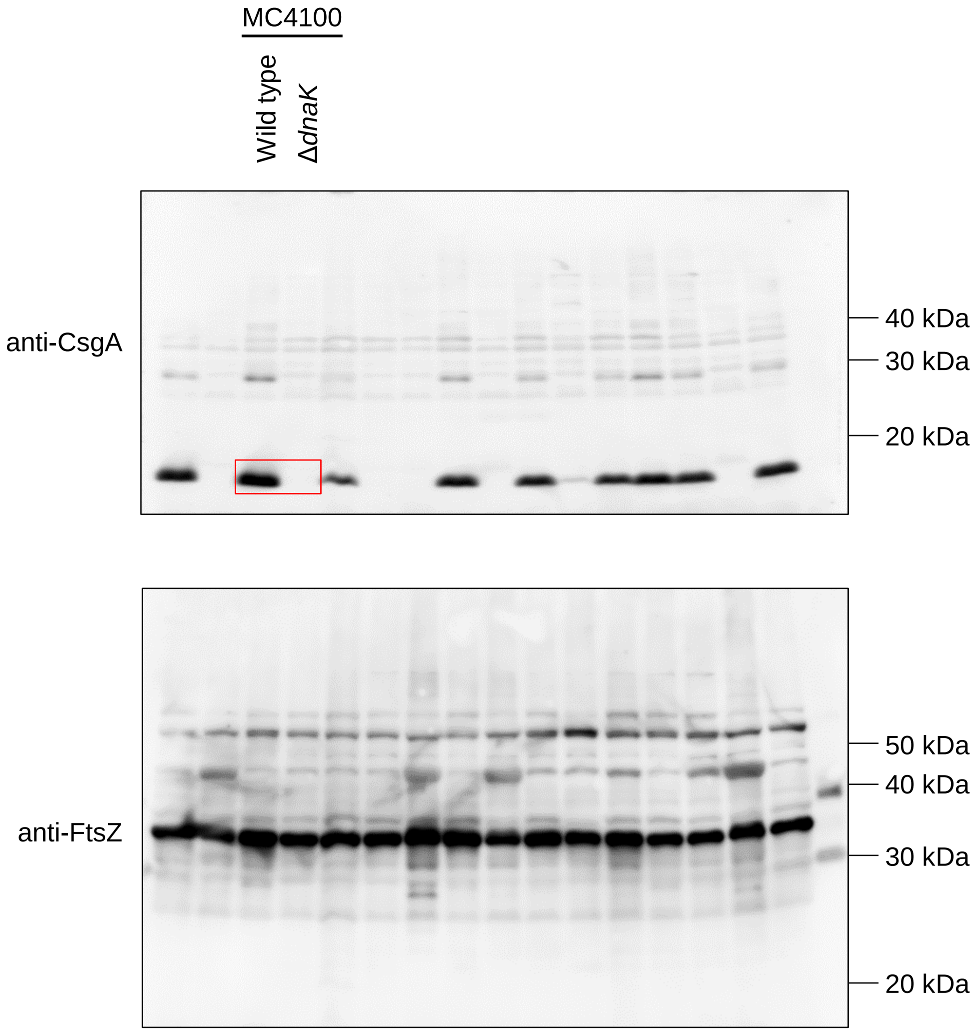

Supplementary Figure 2 Full blots for detection of CsgA and FtsZ. Portions (red squares) are used in Figure 1c. The positions of molecular size markers are shown to the right of the blots.

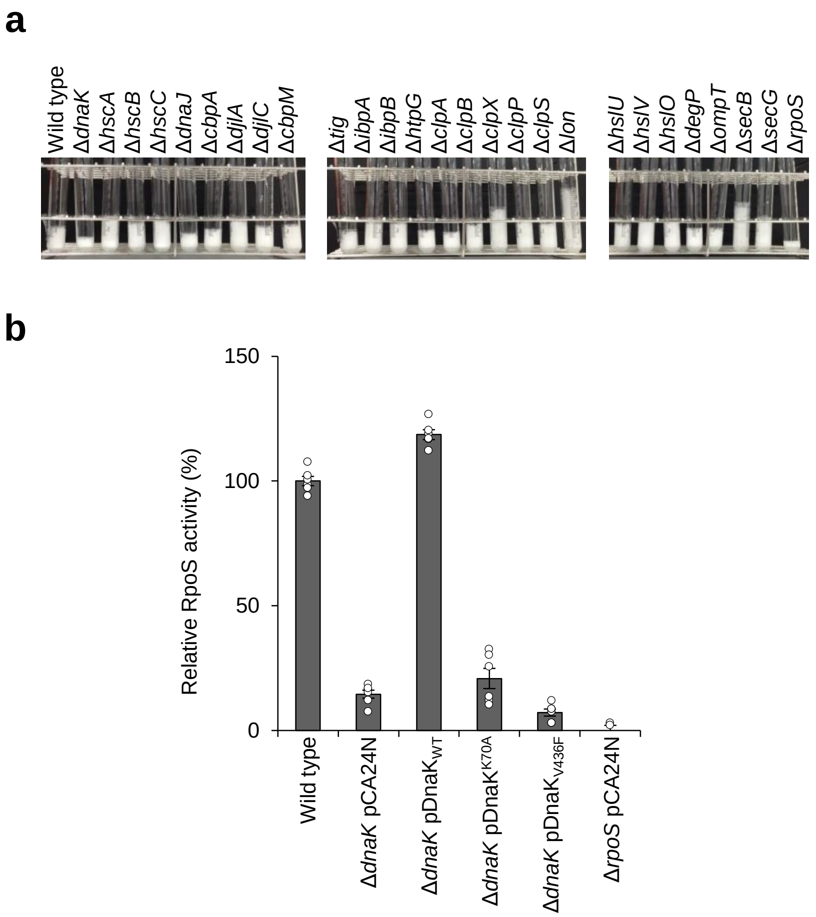

Supplementary Figure 3 Catalase activity of various *E. coli* mutants. **a.** Catalase activities of the BW25113 wild-type and the isogenic mutant strains were analysed in the presence of H_2O_2 and Triton X-100. Pictures of the foam formed by O_2 generation in the catalase reaction are shown. **b.** Relative RpoS activity in the indicated strains was analysed by measuring catalase activity as described in panel **a**. Experiments were repeated at least three times. Means and standard errors and data plots are shown.

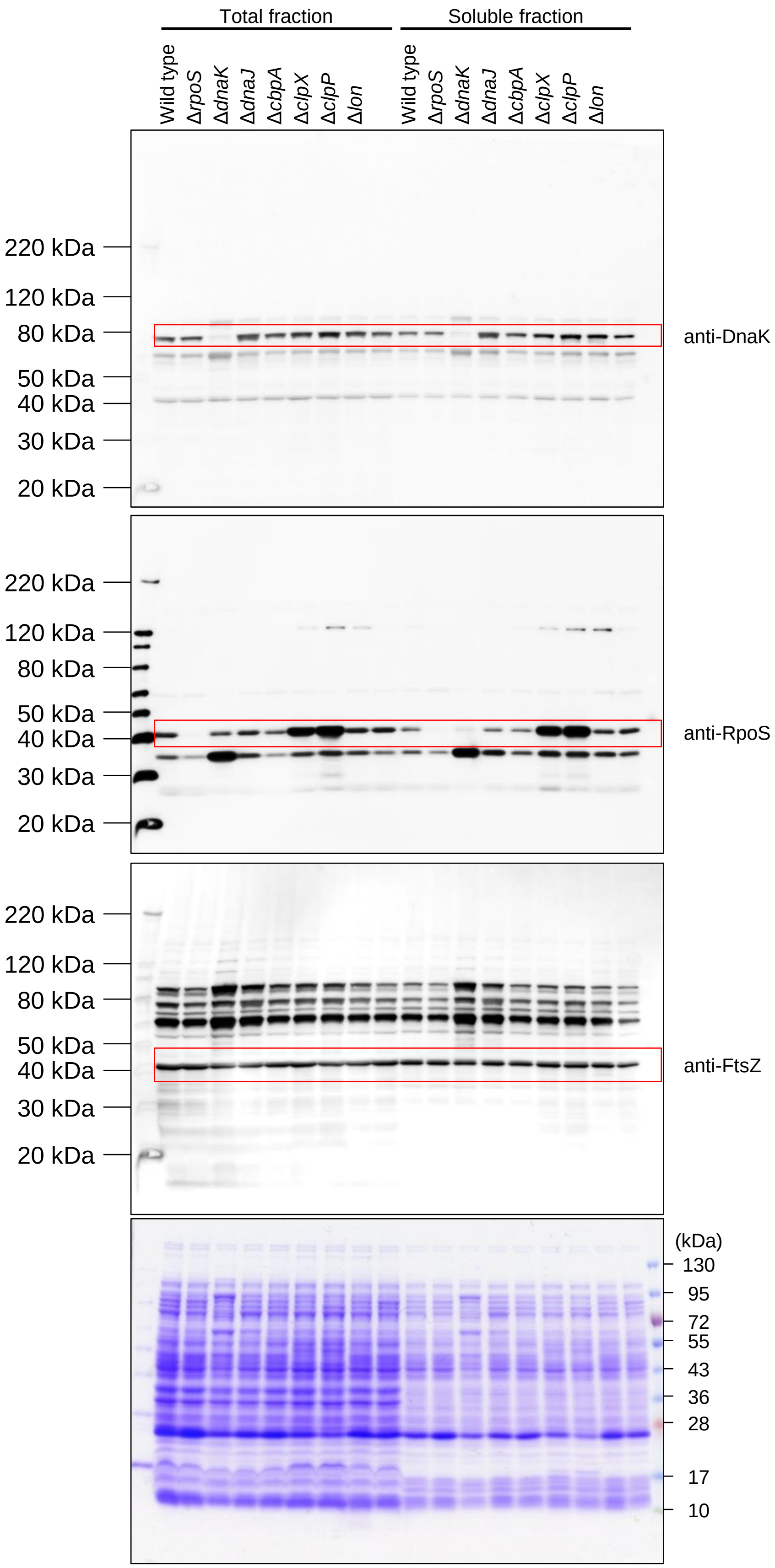

Supplementary Figure 4 Full blots and gels for detection of DnaK, RpoS, FtsZ, and total proteins, respectively. Portions (red squares) are used in Figure 3a. The positions of molecular size markers are shown to the left and right of the blots and gel, respectively.

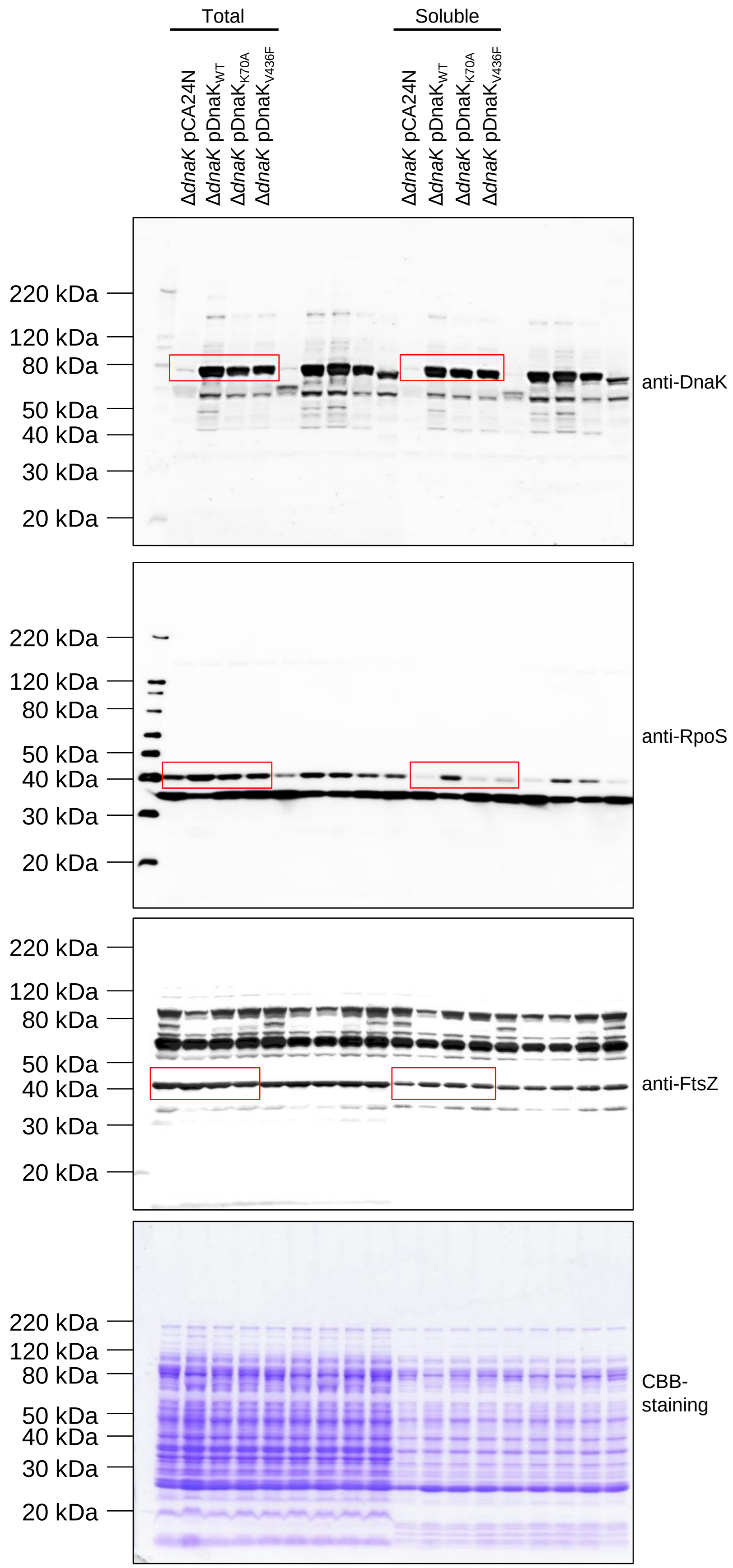

Supplementary Figure 5 Full blots and gels for detection of DnaK, RpoS, FtsZ, and total proteins, respectively. Portions (red squares) are used in Figure 3b. The positions of molecular size markers are shown to the left of the blots and gel.

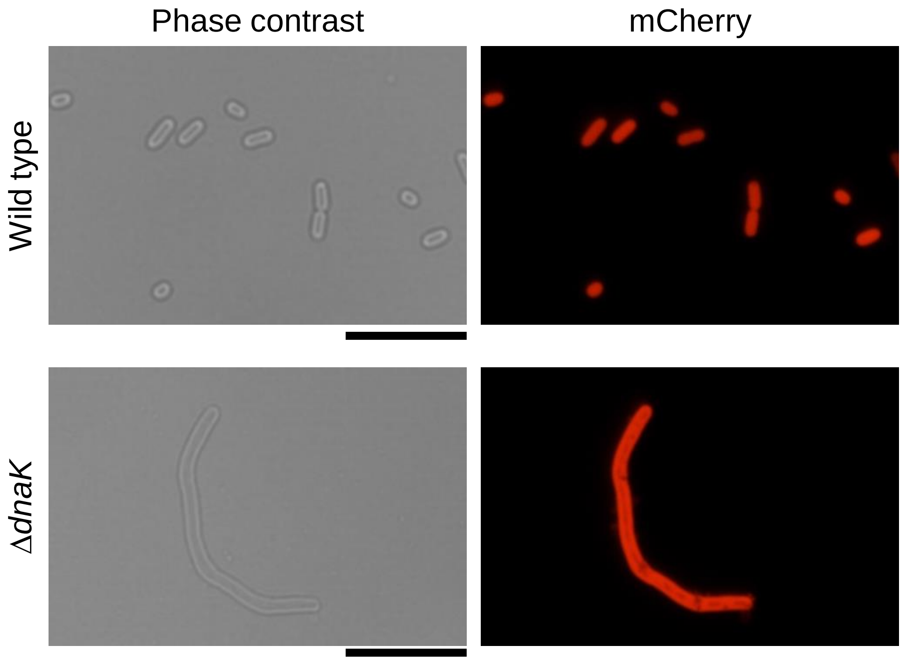

Supplementary Figure 6 Observation of mCherry-expressing cells. Wild-type and Δ*dnaK* cells harboring pCA24-mCherry were observed with fluorescence microscopy. Scales, 10 μm.

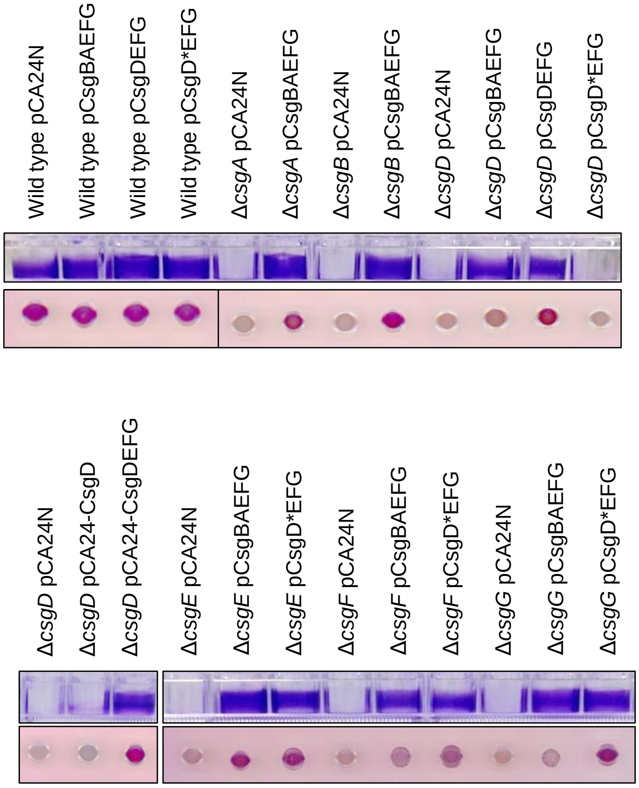

Supplementary Figure 7 Effects of co-expression of *csgDEFG* and *csgBAEFG* on the biofilm formation and curli production in the respective *csg* mutants. Biofilm and curli production of the *E. coli* wild-type and *csg* mutant strains harboring the indicated plasmids were analysed by crystal violet staining and CR-binding assay as shown in Figure 1.

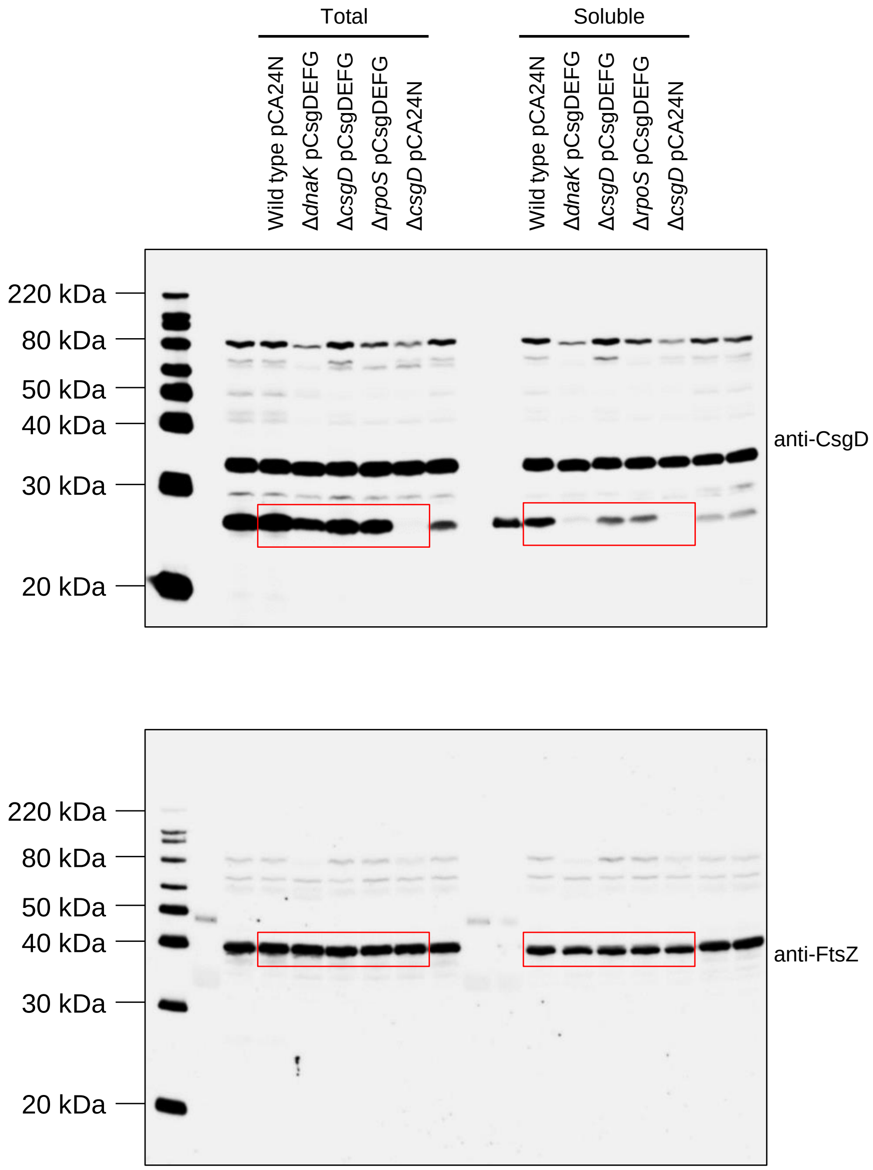

Supplementary Figure 8 Full blots and gels for detection of CsgD and FtsZ. Portions (red squares) are used in Figure 4b. The positions of molecular size markers are shown to the left of the blots.

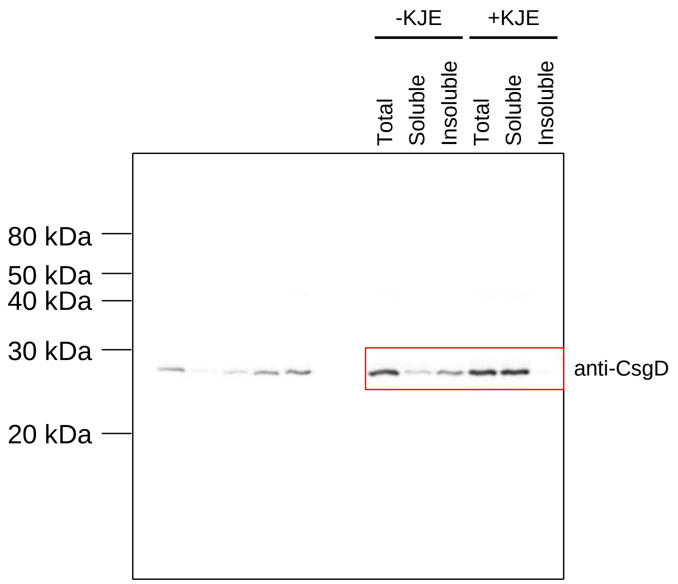

Supplementary Figure 9 Full blots and gels for detection of CsgD. A portion (red squares) is used in Figure 4c. The positions of molecular size markers are shown to the left of the blot.

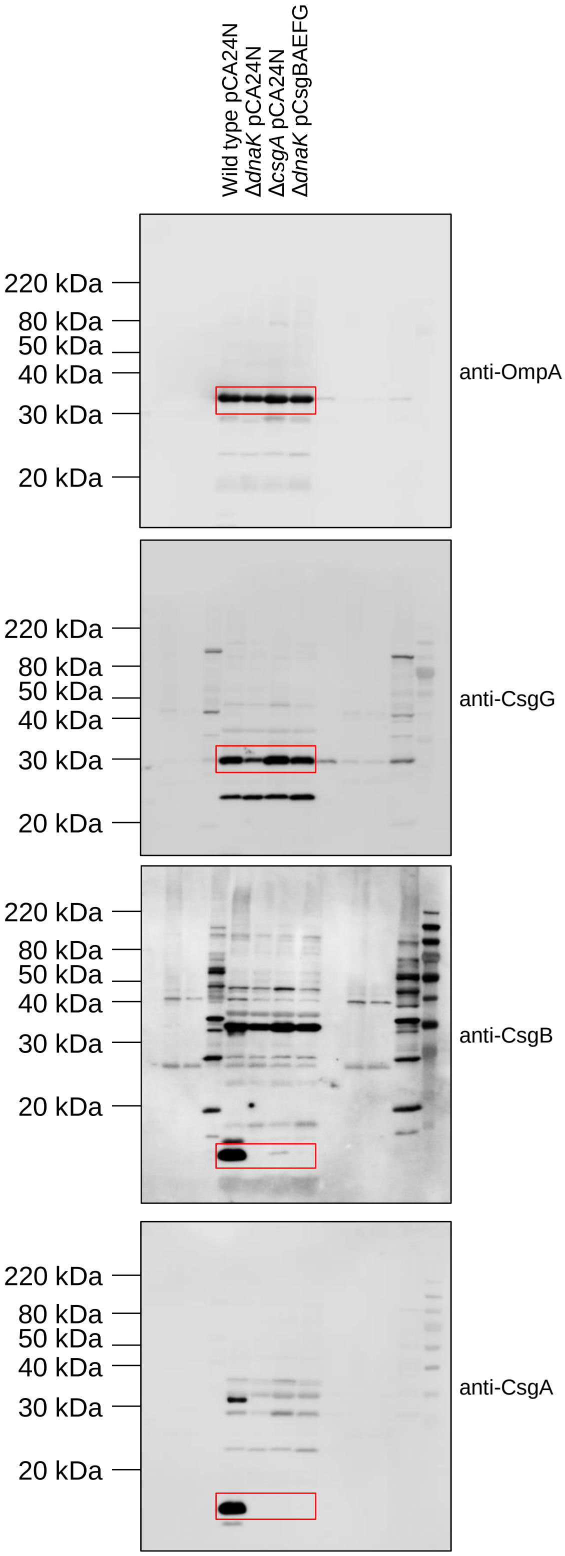

Supplementary Figure 10 Full blots and gels for detection of OmpA, CsgG, CsgB, and CsgA. Portions (red squares) are used in Figure 5c. The positions of molecular size markers are shown to the left of the blots.

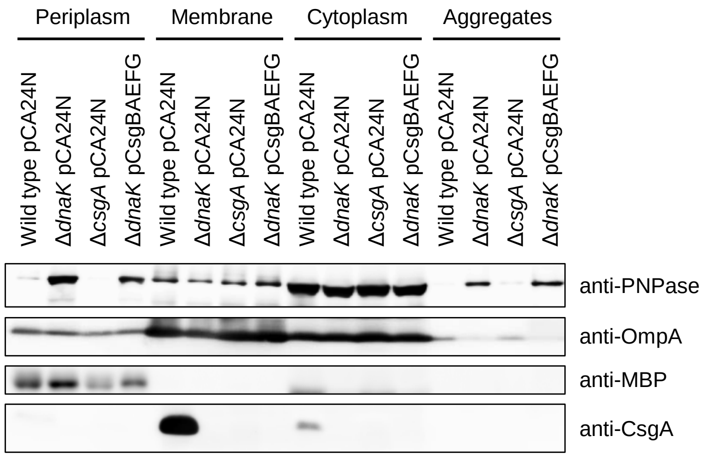

Supplementary Figure 11 Cell fractionation of *E. coli*. After cell fractionation of the indicated, CsgA was detected by immunoblotting to check the cellular localization. To check accuracy of the fractionation, PNPase, OmpA, and MBP were also detected by immunoblotting as cytoplasmic, membrane, and periplasmic proteins, respectively.

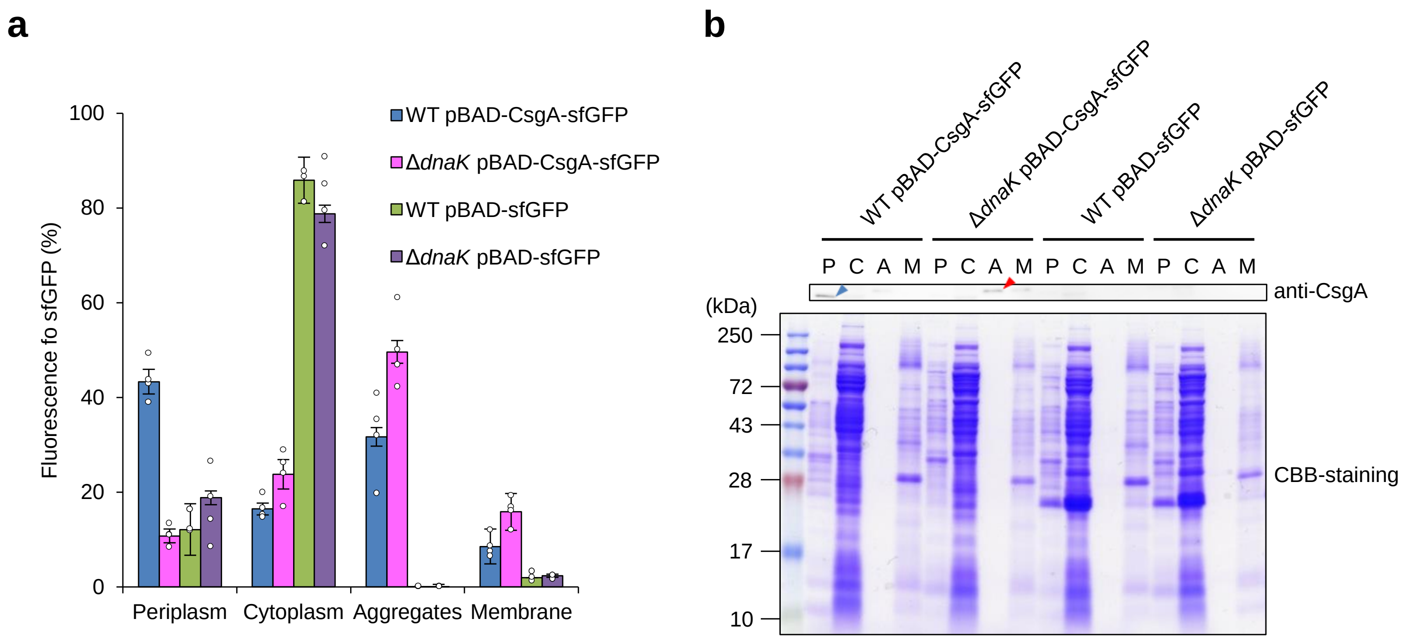

Supplementary Figure 12 Accumulation of CsgA-sfGFP aggregates in the *dnaK* null mutant. **a.** Fluorescence of CsgA-sfGFP in the cellular fractions isolated from the wild-type and Δ*dnaK* strains was measured by fluorophotometer. As a control, sfGFP was also expressed in the strains. Percentages of the fluorescence intensities in each fraction with standard errors and data plots are shown. **b.** The fractionated CsgA-sfGFP was detected by immunoblotting using anti-CsgA antibody. Secretion precursor form and mature form of CsgA-sfGFP were indicated by red and blue arrowheads, respectively. In the wild type, CsgA-sfGFP mainly localized in the periplasm, while, in Δ*dnaK*, it accumulated as aggregates in the cytoplasm. In contrast, sfGFP localized as a soluble form in the cytoplasm of both strains. P: periplasm, C: cytoplasm, A: aggregates, M: membrane. The positions of molecular size markers are shown to the left of the gel.

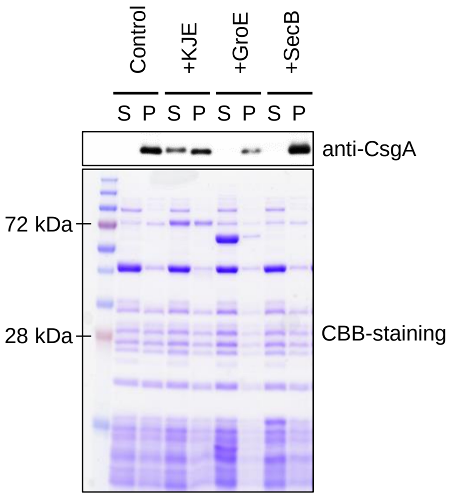

Supplementary Figure 13 DnaK maintains CsgA as a soluble form in vitro. CsgA was synthesized by the cell-free translation system in the absence and presence of DnaK-DnaJ-GrpE (KJE), GroEL/ES (GroE), and SecB. Proteins were separated into the supernatant (S) and pellet (P) fractions by centrifugation and CsgA was detected by immunoblotting. An SDS-PAGE gel was also stained with CBB. The positions of molecular size markers are shown to the left of the gel.

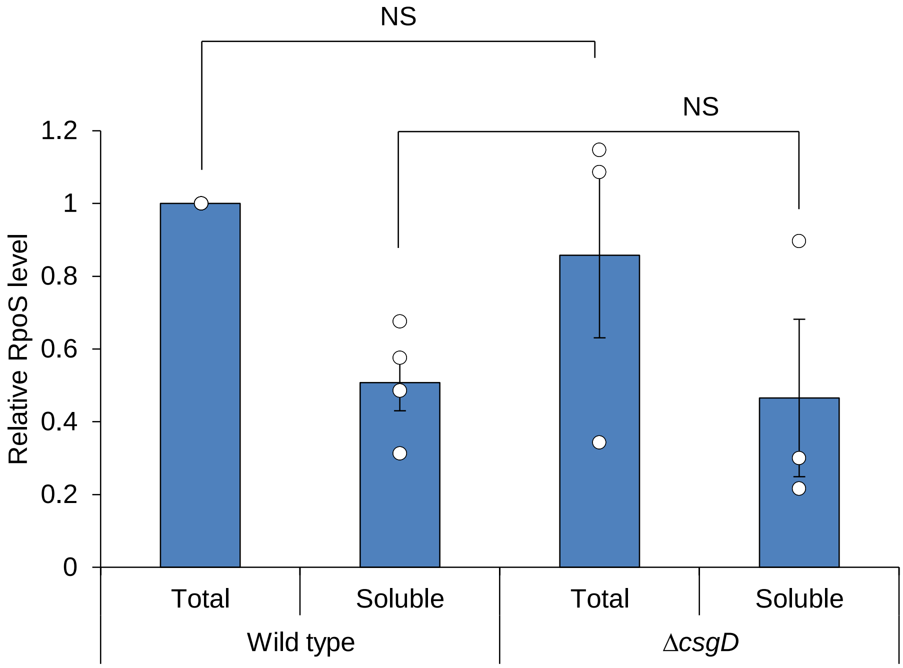

Supplementary Figure 14 RpoS levels in wild type and Δ*csgD*. Band intensities of RpoS in Western blotting data (e.g., Figure 3a) are quantified with Image Quant LAS400 Image Analyzer. Means and standard errors and data plots are shown. NS, not significant.

Supplementary Table 1. Strains and plasmids used in this study

Strains and plasmids	Description	References
***E. coli* strains**		
DH5α	*F-, Φ80dlacZΔM15, Δ(lacZYA-argF)U169, deoR, recA1, endA1, hsdR17(rK-, mK+), phoA, supE44, λ-, thi-1, gyrA96, relA1*	Toyobo
JM109	*recA1 endA1 gyrA96 thi hsdR17(rK− mK+) e14− (mcrA−) supE44 relA1 Δ(lac-proAB)/F'[traD36 proAB+ lacI^{q} lacZΔM15]*	Promega
BL21(DE3)	F^{-} *ompT hsdS*$_B$(*r*$_B^{-}$ *m*$_B^{-}$) *gal(λcI857 ind1 Sam7 nin5 lacUV5-T7gene1) dcm* (DE3)	Promega
MC4100	F^{-} *araDl39* Δ(*argF*$^{-}$*lac)U169 rpsL150 relA1 deoCl ptsF25 rpsR flbB5301*	5
BM271	MC4100 Δ*dnaK52*::Cm^{R}	6
BW25113	K-12 wild type strain of Keio collection	7
JW0013-KC	BW25113 Δ*dnaK*::Km^{R}	7
JW0014-KC	BW25113 Δ*dnaJ*::Km^{R}	7
JW0052-KC	BW25113 Δ*surA*::Km^{R}	7
JW0054-KC	BW25113 Δ*djlA*::Km^{R}	7
JW0157-KC	BW25113 Δ*degP*::Km^{R}	7
JW0426-KC	BW25113 Δ*tig*::Km^{R}	7
JW0427-KC	BW25113 Δ*clpP*::Km^{R}	7
JW0428-KC	BW25113 Δ*clpX*::Km^{R}	7
JW0429-KC	BW25113 Δ*lon*::Km^{R}	7
JW0462-KC	BW25113 Δ*htpG*::Km^{R}	7
JW0554-KC	BW25113 Δ*ompT*::Km^{R}	7

JW0644-KC	BW25113 Δ*djlC*::Km^R	7
JW0645-KC	BW25113 Δ*hscC*::Km^R	7
JW0865-KC	BW25113 Δ*clpS*::Km^R	7
JW0866-KC	BW25113 Δ*clpA*::Km^R	7
JW0984-KC	BW25113 Δ*cbpM*::Km^R	7
JW0985-KC	BW25113 Δ*cbpA*::Km^R	7
JW1020-KC	BW25113 Δ*csgG*::Km^R	7
JW1021-KC	BW25113 Δ*csgF*::Km^R	7
JW1022-KC	BW25113 Δ*csgE*::Km^R	7
JW1023-KC	BW25113 Δ*csgD*::Km^R	7
JW1024-KC	BW25113 Δ*csgB*::Km^R	7
JW1025-KC	BW25113 Δ*csgA*::Km^R	7
JW1058-KC	BW25113 Δ*flgM*::Km^R	7
JW1063-KC	BW25113 Δ*flgE*::Km^R	7
JW1732-KC	BW25113 Δ*spy*::Km^R	7
JW1880-KC	BW25113 Δ*flhC*::Km^R	7
JW1881-KC	BW25113 Δ*flhD*::Km^R	7
JW1908-KC	BW25113 Δ*fliC*::Km^R	7
JW1909-KC	BW25113 Δ*fliD*::Km^R	7
JW1921-KC	BW25113 Δ*fliE*::Km^R	7
JW2510-KC	BW25113 Δ*hscA*::Km^R	7

JW2511-KC	BW25113 Δ*hscB*::Km^R	7
JW2573-KC	BW25113 Δ*clpB*::Km^R	7
JW2861-KC	BW25113 Δ*dsbC*::Km^R	7
JW3142-KC	BW25113 Δ*secG*::Km^R	7
JW3309-KC	BW25113 Δ*fkpA*::Km^R	7
JW3478-KC	BW25113 Δ*hdeA*::Km^R	7
JW3584-KC	BW25113 Δ*secB*::Km^R	7
JW3663-KC	BW25113 Δ*ibpB*::Km^R	7
JW3664-KC	BW25113 Δ*ibpA*::Km^R	7
JW3832-KC	BW25113 Δ*dsbA*::Km^R	7
JW3902-KC	BW25113 Δ*hslU*::Km^R	7
JW3903-KC	BW25113 Δ*hslV*::Km^R	7
JW4230-KC	BW25113 Δ*hfq*::Km^R	7
JW4277-KC	BW25113 Δ*fimA*::Km^R	7
JW4283-KC	BW25113 Δ*fimH*::Km^R	7
JW5182-KC	BW25113 Δ*dsbB*::Km^R	7
JW5437-KC	BW25113 Δ*rpoS*::Km^R	7
JW5692-KC	BW25113 Δ*hslO*::Km^R	7
**Plasmid**		
pET-15b	pET vector with N-terminal His_6-tag, Ap^R	Novagen
pET-28b	pET vector with N-terminal His_6-tag, Km^R	Novagen

pBAD33	Arabinose inducible expression vector	Clontech
pBAD/Myc-His B	Arabinose inducible expression vector with C-terminal Myc-His_6-tag	Life Technologies
pBAD/SS01	Ara promoter, p15A *ori* ; Cm^R, Ap^R	This study
pBAD/SS02	Ara promoter, p15A *ori* ; Ap^R	This study
pCA24N	Empty vector of ASKA clone, *lac* promter ; pBR322*ori* ; Cm^R	8
pCold I	Cold shock promoter ; His_6-tag gene ; ColE1*ori* ; Ap^R	Takara
$pDnaK_{WT}$	*dnaK* cloned in pCA24N, N-terminal His_6-tag, ASKA clone	8
$pDnaK_{K70A}$	N-terminally His_6-tagged $DnaK_{K70A}$ cloned in pCA24N	This study
$pDnaK_{V436F}$	N-terminally His_6-tagged $DnaK_{V436F}$ gene cloned in pCA24N	This study
pRpoS	*rpoS* cloned in pCA24N	This study
pRpoS-mCherry	*rpoS::mcherry* cloned in pCA24N	This study
pCA24-mCherry	*mcherry* cloned in pCA24N	This study
pASKA-CsgD	*csgD* cloned in pCA24N, N-terminal His_6-tag, ASKA clone	8
pCsgD	*csgD* cloned in pCA24N	This study
pCsgDEFG	*csgDEFG* cloned in pCA24N	This study
pCsgD*EFG	DNA-binding domain coding region was deleted from *csgD* in pCsgDEFG	This study
pCsgBAEFG	*csgBA* and *csgEFG* tandemly cloned in pCA24N	This study
pCsgA-His	*csgA* cloned in pCA24N	This study
pBAD-sfGFP	*sfgfp* cloned in pBAD/SS02	This study
pBAD-CsgA-sfGFP	*csgA* cloned in pBAD-sfGFP	This study
pBAD-CsgBA-sfGFP	*csgBA* cloned in pBAD-sfGFP	This study

pBAD-$CsgBA_{1–20}$-sfGFP	*csgB* and *$csgA_{1–20}$* cloned in pBAD-sfGFP	This study
pBAD-$CsgBA_{1–42}$-sfGFP	*csgB* and *$csgA_{1–42}$* cloned in pBAD-sfGFP	This study
pBAD-$CsgBA_{21–42}$-sfGFP	*csgB* and *$csgA_{21–42}$* cloned in pBAD-sfGFP	This study
pBAD-$CsgBA_{21–151}$-sfGFP	*csgB* and *$csgA_{21–151}$* cloned in pBAD-sfGFP	This study
pBAD-$CsgBA_{43–151}$-sfGFP	*csgB* and *$csgA_{43–151}$* cloned in pBAD-sfGFP	This study
pBAD-$CsgBA_{slowgo}$-sfGFP	*csgB* and *$csgA_{slowgo}$* caring Q49A/N54A/Q139A/N144A cloned in pBAD-sfGFP	This study
pBAD-CsgB-mCherry/CsgA-sfGFP	*mcherry* cloned in pBAD-CsgBA-sfGFP	This study
pBAD-CsgD-Myc-His	*csgD* cloned in pBAD/SS02, C-terminally Myc-His_6-tag	This study
pET15-FtsZ	*ftsZ* cloned in pET-15b	9
pET28-PNP	*pnp* cloned in pET-28b	10
pCold-SecB	*secB* cloned in pCold I, N-terminal His_6-tag	This study

[a]Ap^R, ampicillin-resistance; Cm^R, chloramphenicol-resistance; Km^R, kanamycin-resistance.

Supplementary Table 2. Oligonucleotide primers used in this study

Primers	Sequence (5′ to 3′)[a]	Description
dnaK-K70A-F	ACACTCTGTTTGCGATTGCACGCCTGATTGGTCG	For introduction of the substitution of Lys-70 to Ala in DnaK, forward primer
dnaK-K70A-R	CGACCAATCAGGCGTGCAATCGCAAACAGAGTGT	For introduction of the substitution of Lys-70 to Ala in DnaK, reverse primer
dnaK-V436F-F	GACAACCAGTCTGCGTTCACCATCCATGTGCTGC	For introduction of the substitution of Val-436 to Phe in DnaK, forward primer
dnaK-V436F-R	GCAGCACATGGATGGTGAACGCAGACTGGTTGTC	For introduction of the substitution of Val-436 to Phe in DnaK, reverse primer
pCA24N-Art-F	GTCGACCTGCAGCCAAGCTTAATTAGC	For linearization of the empty vector pCA24N, forward primer
pCA24N-Art-R	CAATTTCACACAGAATTCATTAAAGAGGAGAAATTAACTATG	For linearization of the empty vector pCA24N, reverse primer
rpoS-Art-F	GAGAAATTAACTATGATGAGTCAGAATACGCTGAAAGTTCATGATTTAAATG	For cloning of the intact *rpoS* gene into pCA24N, forward primer
rpoS-Art-R	TGGCTGCAGGTCGACTTACTCGCGGAACAGCGCTTCGATATTC	For cloning of the intact *rpoS* gene into pCA24N, reverse primer
rpoS-mcherry-R	TGGCTGCAGGTCGACTTACTTGTACAGCTCGTCCATGCCGC	For cloning of the r*poS-mcheryy* gene into pCA24N, reverse primer
mcherry-inv-F	GAGAAATTAACTATGAAGGGCGAGGAGGATAACATGGCCATCATC	For deletion of rpoS from pRpoS-mCherry, forward primer
mcherry-inv-R	ATCCTCCTCGCCCTTCATAGTTAATTTCTCCTCTTTAATGAATTCTGTGTGAAATTGTTATCC	For deletion of rpoS from pRpoS-mCherry, reverse primer
csgD-Art-F	GAGAAATTAACTATGTTTAATGAAGTCCATAGTATTCATGGTCATAC	For cloning of *csgD* into pCA24N, forward primer
csgD-Art-R	TGGCTGCAGGTCGACTTATCGCCTGAGGTTATCGTTTG	For cloning of *csgD* into pCA24N, reverse primer
csgG-Art-R	TGGCTGCAGGTCGACTCAGGATTCCGGTGGAACCGAC	For cloning of *csgDEFG* and *csgBAEFG* into pCA24N, reverse primer
csgB-Art-F	GAGAAATTAACTATGAAAAACAAATTGTTATTTATGATGTTAACAATACTGGGTGCG	For cloning of *csgBAEFG* into pCA24N, forward primer
csgA-Art-R	TTAGTACTGATGAGCGGTCGCGTTGTTAC	For cloning of *csgBAEFG* into pCA24N, reverse primer
csgA-Art-F	GAGAAATTAACTATGAAACTTTTAAAAGTAGAAGCAATTGCAGCAATCG	For cloning of *csgA-His* into pCA24N, forward primer
csgA-His5-Art-R	TGGCTGCAGGTCGACTTAATGATGATGATGATGGTACTGATGAGCGGTCGCGTTGTTACC	For cloning of *csgA-His* into pCA24N, reverse primer
csgE-Art-F	GCTCATCAGTACTAAACACAAGCGGTTTCCTGGGCAAA	For cloning of *csgBAEFG* into pCA24N, forward primer
csgD*-F	CGTATCGGCGCGTCTAATCTTTTCAAGAAG	For deletion of the DNA-binding domain coding region from

		pCsgDEFG, forward primer
csgD*-R	CTTCTTGAAAAGATTAGACGCGCCGATACG	For deletion of the DNA-binding domain coding region from pCsgDEFG, reverse primer
pBAD33-3000-F	ATCCCTTAACGTGAGTTCCGAAGGTAACTGGCTTCAGCAGAGC	For construction of pBAD/SS01, forward primer
pBAD33-1-R	TGATGACGGTGAAAAGTCCATTTGACAGGCACATTATGCATCG	For construction of pBAD/SS01, reverse primer
pBAD/Myc-His-3033-F	TTTTCACCGTCATCACCGAAACGCGCG	For construction of pBAD/SS01, forward primer
pBAD/Myc-His-1959-R	CTCACGTTAAGGGATTTTGGTCATGAGATTATC	For construction of pBAD/SS01, reverse primer
pBAD33-delta-CP-F	GGCGTTTAAGGGCACCAATAACTGCCTTATGGTGAAAGTTGGAACCTCTTACGTGCC	For construction of pBAD/SS02, for deletion of the Cm^R cassette from pBAD/SS01, forward primer
pBAD33-delta-CP-R	GGCACGTAAGAGGTTCCAACTTTCACCATAAGGCAGTTATTGGTGCCCTTAAACGCC	For construction of pBAD/SS02, for deletion of the Cm^R cassette from pBAD/SS01, reverse primer
pBAD-mcs-F	CGAAGCTTTCTAGAACAAAAACTCATCTCAGAAG	For linearization of pBAD/SS02, forward primer
pBAD-mcs-R	GGTTAATTCCTCCTGTTAGCCCAAAAAACGG	For linearization of pBAD/SS02, reverse primer
sfGFP-Art-F	CAGGAGGAATTAACCATGTCGAAAGGCGAAGAACTGTTTACCGG	For cloning of *sfgfp* into pBAD/SS02, forward primer
sfGFP-Art-R	TTCTAGAAAGCTTCGTTTATACAGCTCATCCATACCATGCGTAATCCC	For cloning of *sfgfp* into pBAD/SS02, reverse primer
pBAD-sfGFP-inverse-F	AGCGATTTTATGTCGAAAGGCGAAGAACTGTTTACCGG	For linearization of pBAD-sfGFP, forward primer
pBAD-csgB-Art-F	CAGGAGGAATTAACCATGAAAAACAAATTGTTATTTATGATGTTAACAATACTGGGTGCG	For cloning of *csgBA* into pBAD-sfGFP, forward primer
pBAD-csgA-Art-R	CGACATAAAATCGCTGTACTGATGAGCGGTCGCGTTGTTACC	For cloning of *csgBA* into pBAD-sfGFP, reverse primer
csgA1-42-sfGFP-F	AATAGCGGCCCAAATAGCGATTTTATGTCGAAAGGCGAAGAAC	For construction of pBAD-CsgBA$_{1–42}$-sfGFP, forward primer
csgA1-42-sfGFP-R	CGACATAAAATCGCTATTTGGGCCGCTATTATTACCGCCACC	For construction of pBAD-CsgBA$_{1–42}$-sfGFP, reverse primer
csgA1-20-sfGFP-F	GGTAGCGCTCTGGCAAGCGATTTTATGTCGAAAGGCGAAGAAC	For construction of pBAD-CsgBA$_{1–20}$-sfGFP, forward primer
csgA1-20-sfGFP-R	CGACATAAAATCGCTTGCCAGAGCGCTACCGGAGAATACGATTG	For construction of pBAD-CsgBA$_{1–20}$-sfGFP, reverse primer
csgA43-151-sfGFP-F	GAGGAATTAACCATGTCTGAGCTGAACATTTACCAGTACGGTGG	For construction of pBAD-CsgBA$_{43–151}$-sfGFP, forward primer
csgA43-151-sfGFP-R	AATGTTCAGCTCAGACATGGTTAATTCCTCCTGTTAGCCCAAAAAAC	For construction of pBAD-CsgBA$_{43–151}$-sfGFP, reverse primer
csgA21-151-sfGFP-F	GAGGAATTAACCATGGGTGTTGTTCCTCAGTACGGCGGCGG	For construction of pBAD-CsgBA$_{21–151}$-sfGFP and pBAD-CsgBA$_{21–42}$-sfGFP, forward primer
csgA21-151-sfGFP-R	CTGAGGAACAACACCCATGGTTAATTCCTCCTGTTAGCCCAAAAAAC	For construction of pBAD-CsgBA$_{21–151}$-sfGFP and pBAD-CsgBA$_{21–42}$-sfGFP, reverse primer
Q49A_N54A-F	TACGCATACGGTGGCGGTGCATCTGCACTTGCTCTGCAAACTGATG	For introduction of the substitutions of Gln-49 to Ala and Asn-54 in CsgA, forward primer

Q49A_N54A-R	AGATGCACCGCCACCGTATGCGTAAATGTTCAGCTCAGAATTTGGG	For introduction of the substitutions of Gln-49 to Ala and Asn-54 in CsgA, reverse primer
Q139A_N144A-F	CGTGACTGCAGTTGGCTTTGGTGCAAACGCGACCGCTCATCAGTACAG	For introduction of the substitutions of Gln-139 to Ala and Asn-144 in CsgA, forward primer
Q139A_N144A-R	GCGTTTGCACCAAAGCCAACTGCAGTCACGTTGACGGAGGAGTTAGATG	For introduction of the substitutions of Gln-139 to Ala and Asn-144 in CsgA, reverse primer
csgB-csgA-inter-F	TAATTTCCATTCGACTTTTAAATCAATCCGATGGG	For construction of pBAD-CsgB-mCherry/CsgA-sfGFP, forward primer
csgB-csgA-inter-R	ACGTTGTGTCACGCGAATAGCCATTTG	For construction of pBAD-CsgB-mCherry/CsgA-sfGFP, reverse primer
csgB-mcherry-Art-F	TCGCGTGACACAACGTAAGGGCGAGGAGGATAACATGGCC	For construction of pBAD-CsgB-mCherry/CsgA-sfGFP, forward primer
csgB-mcherry-Art-R	GTCGAATGGAAATTACTTGTACAGCTCGTCCATGCCGC	For construction of pBAD-CsgB-mCherry/CsgA-sfGFP, reverse primer
secB-ArtF	GAAGGTAGGCATATGTCAGAACAAAACAACACTGAAATGACTTTCC	Cloning of *secB* into pCold I, forward primer
secB-Art-R	AGAGATTACCTATCTTCAGGCATCCTGATGTTCTTCAGTACCTTC	Cloning of *secB* into pCold I, reverse primer
Pure-Niwa-F	GGCCTAATACGACTCACTATAGGAGAAATCATAAAAAATTTATTTGCTTTGTGAGCGG	For in vitro translation using PURE System, forward primer
Pure-CsgD-R	GTTATTGCTCAGCGGTTAGCGGCCGCATAGGCC	For in vitro translation of CsgD using PURE System, reverse primer
csgB-UTR-Alexa-F	GATAACAGCGTATTTACGTGGGTTTTAATACTTTGG	For gel-shift analysis, amplification of Alexa488-labeled *csgB* promoter forward primer
csgB-UTR-R	ACGCAACCTGTATTTTGTTAACGCTGC	For gel-shift analysis, amplification of Alexa488-labeled *csgB* promoter reverse primer
RT-csgA-F	TCTGGCAGGTGTTGTTCCTC	For RT-PCR, forward primer
RT-csgA-R	CCACCACCATGCTGGGTAAT	For RT-PCR, reverse primer
RT-csgD-F	TCTCGTTATTAGACGCGCCG	For RT-PCR, forward primer
RT-csgD-R	CAATGGATTGCAAGGCGTCC	For RT-PCR, reverse primer

[a] Underlines, mutation sites.

SUPPLEMENTARY DISCUSSION

Biofilm quantification data

We compared growth of *E. coli* BW25113 wild type and Δ*dnaK* on LB plates and LB liquid media and found that there was no remarkable difference between at least these strains (Fig. 1f and data not shown). Therefore, normalization of biofilm biomass by cell growth (optical density or colony forming units) was not performed in this study.

Microarray data

We summarized microarray data of previously reported RpoS-regulated genes[1] and RpoH-regulated genes[2] in Fig. 2c. Full data set is also shown in Supplementary Data 1. Expression of many, but not all, genes positively regulated by RpoS was decreased in Δ*dnaK* compared to wild type. Given that a few soluble RpoS is present in Δ*dnaK* (Fig. 3b), some of the unaffected genes may be regulated by small but enough amount of active RpoS. It is also likely that expression of these genes may be independent on RpoS under the conditions tested in this study (in YESCA medium at 30°C for 48 h), since culture conditions affect the RpoS-dependency[1,3].

Cellular localization and aggregation analyses using fluorescent proteins

To visualize cellular localization and protein aggregates, we used mCherry and sfGFP. As shown in Fig. 6b and Supplementary Fig. 6, no aggregation of these proteins was observed. Therefore, aggregates of RpoS-mCherry and CsgA-sfGFP derivatives formed in *dnaK* cells were likely not due to the fusion of these fluorescent proteins. The fluorescent fusion proteins can be prone to aggregation and the foci visualized may not be the real foci but instead are demonstrating the underlying trend that the Δ*dnaK* strain has more aggregates. The RpoS data is supported by fractionation (Fig. 3).

SUPPLEMENTARY METHODS

Protein purification

C-terminal Myc-His tagged CsgD (CsgD-Myc-His) was purified as follows. *E. coli* BL21 (DE3) harboring pBAD-CsgD-Myc-His was cultured in 20 mL LB medium containing 100 µg/mL ampicillin overnight at 30°C with shaking at 150 rpm. Cultures were diluted in 1-L LB medium supplemented with 100 µg/mL ampicillin and incubated at 30°C with shaking at 100 rpm. When optical density at 660 nm reached 0.5, the culture was supplemented with 0.02% arabinose followed by overnight incubation at 15°C. After centrifugation at 6,000 × *g* for 20 min at 4°C, harvested cells were resuspended in 50 mL buffer A supplemented with protease inhibitor cocktail. After sonication on ice, cell lysates were centrifuged at 9,400 × *g* for 30 min at 4°C, and the supernatant was loaded onto a 1-mL bed volume of TALON resin, which was washed with buffer A supplemented with 5 mM imidazole; recombinant proteins were then eluted with 250 mM imidazole. Since the eluted fractions formed aggregates, insoluble proteins were pelleted by centrifugation at 20,000 × *g* for 10 min at 4°C and washed with buffer D [10 mM Tris-HCl (pH 8.0), 1% (w/v) Nonidet P-40, and 500 mM NaCl]. Following centrifugation at 20,000 × *g* for 10 min at 4°C, insoluble proteins were incubated overnight at room temperature in buffer E [10 mM Tris-HCl (pH 8.0), and 8 M urea], centrifuged at 20,000 × *g* for 10 min at room temperature, and solubilized in buffer F [50 mM Tris-HCl (pH 8.0), 6 M Guanidine-HCl (Gdn-HCl), and 200 mM NaCl] at room temperature for 1 h before purification using TALON resin under denature conditions. The resin was washed with buffer F supplemented with 10 mM imidazole, and recombinant proteins were eluted using buffer F supplemented with 250 mM imidazole. Eluted fractions were pooled and dialyzed overnight at 4°C against buffer G [10 mM Tris-HCl (pH 8.0), 10 mM $MgCl_2$, 100 mM KCl, and 1 mM DTT] supplemented with 6 M Gdn-HCl, overnight at 4°C in buffer G supplemented with 2 M Gdn-HCl and 400 mM L-arginine-HCl, for 5 h at 4°C in buffer G supplemented with 1 M Gdn-HCl and 400 mM L-arginine-HCl, and overnight at 4°C in buffer G supplemented with 400 mM L-arginine-HCl and 20% glycerol using the Slide-A-Lyzer Dialysis Cassette (Thermo Fisher Scientific). Purified CsgD-Myc-His was quantified with the

Bradford Assay Kit.

N-terminally His_6-tagged SecB (His-SecB) was expressed in *E. coli* BL21(DE3). Cells harboring pCold-SecB were grown at 30°C in LB medium containing 100 μg/mL ampicillin, and expression of His-SecB was induced by the adding IPTG (0.1 mM) and incubating overnight at 15°C. Cells from 2-L culture were harvested by centrifugation and resuspended in 50 mL buffer A supplemented with a protease inhibitor cocktail. After sonication on ice, cell lysates were centrifuged at 9,000 × *g* for 30 min at 4°C, and the supernatant was loaded onto a 2-mL bed volume of TALON resin that was washed with buffer A supplemented with 5 mM imidazole. Recombinant proteins were eluted using 250 mM imidazole. Eluted fractions were dialyzed against buffer B using Slide-A-Lyzer Dialysis Cassette and purified by chromatography using a HiTrap Q column (GE Healthcare) and a 0–1,000 mM NaCl gradient in buffer B. Each fraction containing His-SecB was pooled and further purified by size exclusion chromatography (Superdex G-75 Increase 10/300 GL; GE Healthcare) in buffer C. Purified His-SecB was pooled and quantified using a Bradford Assay Kit.

Catalase activity assay

Catalase activity in overnight cultures was measured as previously described[4] with minor modifications. Bacteria were grown in 5 mL LB medium without supplementation or supplemented with 30 μg/mL chloramphenicol at 30°C for 16 h with shaking under aerobic conditions. Bacterial cells were harvested by centrifugation at 8,000 × *g* for 20 min and suspended in 1 mL PBS supplemented with 1% Triton X-100. A 200 μL volume of the solutions was mixed with 200 μL undiluted hydrogen peroxide (30%) and incubated at room temperature for 5 min before measuring the height of O_2-forming foam.

SUPPLEMENTARY REFERENCES

1. Vijayakumar, S. R., Kirchhof, M. G., Patten, C. L. & Schellhorn, H.E. RpoS-regulated genes of *Escherichia coli* identified by random *lacZ* fusion mutagenesis. *J. Bacteriol.* **186**, 8499−8507 (2004).
2. Guisbert, E., Yura, T., Rhodius, V. A. & Gross, C. A. Convergence of molecular,

modeling, and systems approaches for an understanding of the *Escherichia coli* heat shock response. *Microbiol. Mol. Biol. Rev.* **72**, 545−554 (2008).

3. Dong, T., Kirchhof, M. G. & Schellhorn, H. E. RpoS regulation of gene expression during exponential growth of *Escherichia coli* K12. *Mol. Genet. Genomics.* **279**, 267−277 (2008).
4. Iwase, T., Tajima, A., Sugimoto, S., Okuda, K., Hironaka, I., Kamata, Y., Takada, K. & Mizunoe, Y. A simple assay for measuring catalase activity: a visual approach. *Sci. Rep.* **3**, 3081 (2013).
5. Casadaban, M. J. Transposition and fusion of the lac genes to selected promoters in *Escherichia coli* using bacteriophage lambda and Mu. *J. Mol. Biol.* **104**, 541−555 (1976).
6. Bukau, B. & Walker, G. C. Cellular defects caused by deletion of the *Escherichia coli dnaK* gene indicate roles for heat shock protein in normal metabolism. *J. Bacteriol.* **171**, 2337−2346 (1989).
7. Baba, T., Ara, T., Hasegawa, M., Takai, Y., Okumura, Y., Baba, M., Datsenko, K. A., Tomita, M., Wanner, B.L. & Mori, H. Construction of *Escherichia coli* K-12 in-frame, single-gene knockout mutants: the Keio collection. *Mol. Syst. Biol.* **2**, 2006.0008 (2006).
8. Kitagawa, M., Ara, T., Arifuzzaman, M., Ioka-Nakamichi, T., Inamoto, E., Toyonaga, H. & Mori, H. Complete set of ORF clones of *Escherichia coli* ASKA library (a complete set of *E. coli* K-12 ORF archive): unique resources for biological research. *DNA Res.* **12**, 291−299 (2005).
9. Sugimoto, S., Yamanaka, K., Nishikori, S., Miyagi, A., Ando, T. & Ogura, T. AAA+ chaperone ClpX regulates dynamics of prokaryotic cytoskeletal protein FtsZ. *J. Biol. Chem.* **285**, 6648−6657 (2010).
10. Sugimoto, S., Arita-Morioka, K., Mizunoe, Y., Yamanaka, K. & Ogura, T. Thioflavin T as a fluorescence probe for monitoring RNA metabolism at molecular and cellular levels. *Nucleic Acids Res.* **43**, e92 (2015).
