# Supplementary material for: Multitasking of Hsp70 chaperone in the biogenesis of bacterial functional amyloids
Source: Commun Biol. 2018 May 31;1:52. doi: 10.1038/s42003-018-0056-0 (PMC6123696; doi:10.1038/s42003-018-0056-0)
Supplement: Supplementary file 2 — Description of Additional Supplementary Files [file 42003_2018_56_MOESM2_ESM.docx]

**Description of Additional Supplementary Files**

File Name: Supplementary Data 1

Description: Supplementary Data 1. Microarray data represents up- and down-regulated genes in *∆dnaK*, Related to Figure 2
